# Heterogeneous Expression of Nuclear Encoded Mitochondrial Genes Distinguishes Inhibitory and Excitatory Neurons

**DOI:** 10.1523/ENEURO.0232-21.2021

**Published:** 2021-08-07

**Authors:** Meghan E. Wynne, Alicia R. Lane, Kaela S. Singleton, Stephanie A. Zlatic, Avanti Gokhale, Erica Werner, Duc Duong, Jennifer Q. Kwong, Amanda J. Crocker, Victor Faundez

**Affiliations:** 1 Department of Cell Biology, Emory University, Atlanta, GA 30322; 2 Department of Biochemistry, Emory University, Atlanta, GA 30322; 3 Department of Pediatrics, Emory University, Atlanta, GA 30322; 4 Program in Neuroscience, Middlebury College, Middlebury, VT 05753

**Keywords:** GABA, glutamate, mitochondria, mitochondrial ribosome, respiratory chain, solute transporter

## Abstract

Mitochondrial composition varies by organ and their constituent cell types. This mitochondrial diversity likely determines variations in mitochondrial function. However, the heterogeneity of mitochondria in the brain remains underexplored despite the large diversity of cell types in neuronal tissue. Here, we used molecular systems biology tools to address whether mitochondrial composition varies by brain region and neuronal cell type in mice. We reasoned that proteomics and transcriptomics of microdissected brain regions combined with analysis of single-cell mRNA sequencing (scRNAseq) could reveal the extent of mitochondrial compositional diversity. We selected nuclear encoded gene products forming complexes of fixed stoichiometry, such as the respiratory chain complexes and the mitochondrial ribosome, as well as molecules likely to perform their function as monomers, such as the family of SLC25 transporters. We found that the proteome encompassing these nuclear-encoded mitochondrial genes and obtained from microdissected brain tissue segregated the hippocampus, striatum, and cortex from each other. Nuclear-encoded mitochondrial transcripts could only segregate cell types and brain regions when the analysis was performed at the single-cell level. In fact, single-cell mitochondrial transcriptomes were able to distinguish glutamatergic and distinct types of GABAergic neurons from one another. Within these cell categories, unique SLC25A transporters were able to identify distinct cell subpopulations. Our results demonstrate heterogeneous mitochondrial composition across brain regions and cell types. We postulate that mitochondrial heterogeneity influences regional and cell type-specific mechanisms in health and disease.

## Significance Statement

Mitochondria are important organelles for maintaining brain health. The composition of proteins making up mitochondria is essential for their function. Disturbances to mitochondria are thought to contribute to neurodegeneration and neurodevelopmental disorders. These conditions typically affect specific brain regions or cell types. Despite the link between mitochondria and diseases with distinct anatomic and cellular patterns, how mitochondrial composition varies across brain regions and cell types remains poorly explored. Here, we analyze mitochondrial composition in different brain regions and cell types in adult mice, showing composition differs by region and cell lineage. Our work provides a resource of genes enriched in certain cell types or regions that improves our understanding of how mitochondrial composition influences brain function in health and disease.

## Introduction

The mitochondrion is classically depicted as the powerhouse of the cell despite performing a variety of functions outside of ATP production (Spinelli and Haigis, 2018). From a purely bioenergetic perspective, some of these functions are necessary for energy requirements to maintain plasma membrane potential, synaptic activity, and actin cytoskeleton dynamics (Attwell and Laughlin, 2001; Bernstein and Bamburg, 2003; Harris et al., 2012). However, additional roles for mitochondria have been identified in behavior, synaptic plasticity, neuronal migration, neurodevelopment, calcium buffering, lipid metabolism, and cell death (Kann and Kovács, 2007; Mattson et al., 2008; Mann et al., 2021). The requirement of functional mitochondria for neuronal tissue is perhaps best demonstrated by the family of mitochondrial diseases, which share a high prevalence of neurologic symptoms despite being otherwise clinically heterogeneous (Chinnery, 1993; Vafai and Mootha, 2012; Gorman et al., 2016).

Mitochondria are dynamic organelles and vary phenotypically by organ, cell type, and even within the cell (Pagliarini et al., 2008; Aryaman et al., 2018; Fecher et al., 2019; Rath et al., 2021). These differences in phenotypes may emerge because of variation in mitochondrial composition across cell types and/or within a single cell. This concept has been poorly considered and explored to date, as most studies of mitochondrial biology involve bulk purification of mitochondria from diverse organs (Pagliarini et al., 2008; Fecher et al., 2019; Rath et al., 2021). Cell type-specific differences in mitochondrial composition could determine differential cellular susceptibility to neurodevelopmental disorders and neurodegenerative diseases. Here, we address whether mitochondrial composition varies across cell types and brain regions. We take advantage of systems biology gene expression analyses in microdissected brain tissue and single-cell mRNA sequencing (mRNAseq) datasets. We analyzed the transcriptome and proteome in microdissected mouse cortex, hippocampus, and striatum. We focused on the five respiratory chain complexes and the mitochondrial ribosome, as necessary components of mitochondria that have a fixed stoichiometry (Vafai and Mootha, 2012), as well as the SLC25A transporter family, as molecules of variable expression among tissues (Cunningham and Rutter, 2020; Palmieri et al., 2020; Rath et al., 2021). Collectively, this set of genes encompasses 18% of the mitochondria-annotated proteome (Rath et al., 2021). Notably, while the expression of this selected set of nuclear encoded mitochondrial genes produced distinct regional clusters differentiating the cortex, hippocampus, and striatum at the proteome level, analysis of the transcript expression of these nuclear encoded mitochondrial genes could not distinguish between these three different brain regions. However, at the single-cell level, distinct cortical and hippocampal regions could be distinguished by differential expression of mitochondrial ribosome, SLC25A (inner mitochondrial membrane transporters), or individual respiratory chain complex transcripts. Expression of mitochondrial genes could prominently distinguish excitatory and inhibitory neurons, as well as different classes of GABAergic interneurons.

The present study demonstrates that nuclear encoded mitochondrial transcripts and proteins are differentially expressed across brain regions and cell types, informing our understanding of the molecular diversity and heterogeneity within the brain. Our work expands recent findings demonstrating that mitochondria differ in composition among cell populations in the cerebellum (Fecher et al., 2019) and between fast-spiking and regular spiking neurons (Cserép et al., 2018). We postulate that cell lineage-specific mitochondrial composition and metabolism are poised to contribute to the susceptibility of certain cell types to damage and/or cell death in diseases of the nervous system.

## Materials and Methods

### Animals and tissue dissection

Animal husbandry and euthanasia was conducted as approved by our Institutional Animal Care and Use Committees. C57BL/6J male mice (The Jackson Laboratory #000664), six weeks of age, were euthanized with CO_2_ asphyxiation and decapitated. Whole brain was removed, rinsed in ice-cold phosphate buffered saline and placed in a prechilled adult mouse coronal slicing matrix with 1.0-mm slice interval (Zivic catalog #BSMAS001-1). Chilled blades were placed in the matrix channels according to manufactures recommendations and slices laid out on an ice-cold aluminum block for punch microdissection. Hippocampal regions were identified in sections #2 and #3 of the slices corresponding to sections 21–22 of the C57BL/6J Atlas (http://www.mbl.org/atlas170/atlas170_frame.html). Cortex punches were taken adjacent to the hippocampal regions. Striatum was dissected from slice #6 or #7 corresponding to sections 15–16 of the C57BL/6J Atlas. Punches of the brain tissue were taken using a chilled punch set with 1.00-mm diameter punches (Stoelting catalog #57401): six punches were taken from each of the hippocampus, cortex, and striatum brain regions (three from the left hemisphere and three from the right hemisphere; Barr et al., 2004). Punches were ejected, transferred to a microcentrifuge tube using forceps, and flash frozen in liquid nitrogen until processing for RNAseq or mass spectrometry (MS).

### RNAseq

RNA extraction, library construction, and sequencing were done by BGI and are briefly described below. Total RNA was extracted with TRIzol and quality control was performed with the Agilent 2100 Bio analyzer (Agilent RNA 6000 Nano kit) to do the total RNA sample QC: RNA concentration, RIN value, 28S/18S, and the fragment length distribution.

For library construction, poly-A containing mRNA molecules were isolated using poly-T oligo-attached magnetic beads. Following purification, the mRNA was fragmented into small pieces using divalent cations under elevated temperature. The cleaved RNA fragments were copied into first strand cDNA using reverse transcriptase and random primers. This was followed by second strand cDNA synthesis using DNA Polymerase I and RNase H. cDNA fragments underwent addition of a single “A” base and subsequent ligation of the adapter. The products were then purified and enriched with PCR amplification. We quantified the PCR yield by Qubit and pooled samples together to make a single strand DNA circle (ssDNA circle), which gave the final library. DNA nanoballs (DNBs) were generated with the ssDNA circle by rolling circle replication (RCR) to enlarge the fluorescent signals at the sequencing process. The DNBs were loaded into the patterned nanoarrays and pair-end reads of 100 bp were read through on the BGISEQ-500 platform for data analysis. For this step, the BGISEQ-500 platform combined the DNB-based nanoarrays and stepwise sequencing using combinational probe-anchor synthesis sequencing method. On average, we generated ∼5.64 Gb bases per sample. The average mapping ratio with reference genome was 93.47%, the average mapping ratio with gene was 67.04%; 19,972 genes were identified in which 19,972 of them are known genes and 2659 of them are novel genes; 29,781 novel transcripts were identified.

#### Analysis of sequencing reads

The sequencing reads were uploaded to the Galaxy web platform, and we used the public server at https://usegalaxy.org/ to analyze the data (Afgan et al., 2018). FastQC was performed to remove samples of poor quality (Andrews, 2010). All mapping was performed using Galaxy server (v. 21.01) running Hisat2 (Galaxy version 2.1.0+galaxy7), FeatureCounts (Galaxy version 2.0.1), and Deseq2 (Galaxy version 2.11.40.6+galaxy1; Liao et al., 2014; Love et al., 2014; Kim et al., 2015). The Genome Reference Consortium build of the reference sequence (GRCm38) and the GTF files (Ensembl) were used and can be acquired from iGenome (Illumina). Hisat2 was run with the following parameters: paired-end, unstranded, default settings were used except for a GTF file was used for transcript assembly. The aligned SAM/BAM files were processed using Featurecounts (Default settings except used Ensembl GRCm38 GTF file and output for DESeq2 and gene length file). FeatureCounts output files and raw read files are publicly available (GEO with accession GSE140054). The FeatureCounts compiled file is GSE140054_AllTissueFeatureCounts.txt.gz. Gene counts were normalized using DESeq2 (Love et al., 2014) followed by a regularized log transformation. Differential Expression was determined using DESeq2 with the following settings: factors were tissue type, pairwise comparisons across tissues was done, output all normalized tables, size estimation was the standard median ratio, fit type was parametric, outliers were filtered using a Cook’s distance cutoff.

**Figure 1.**
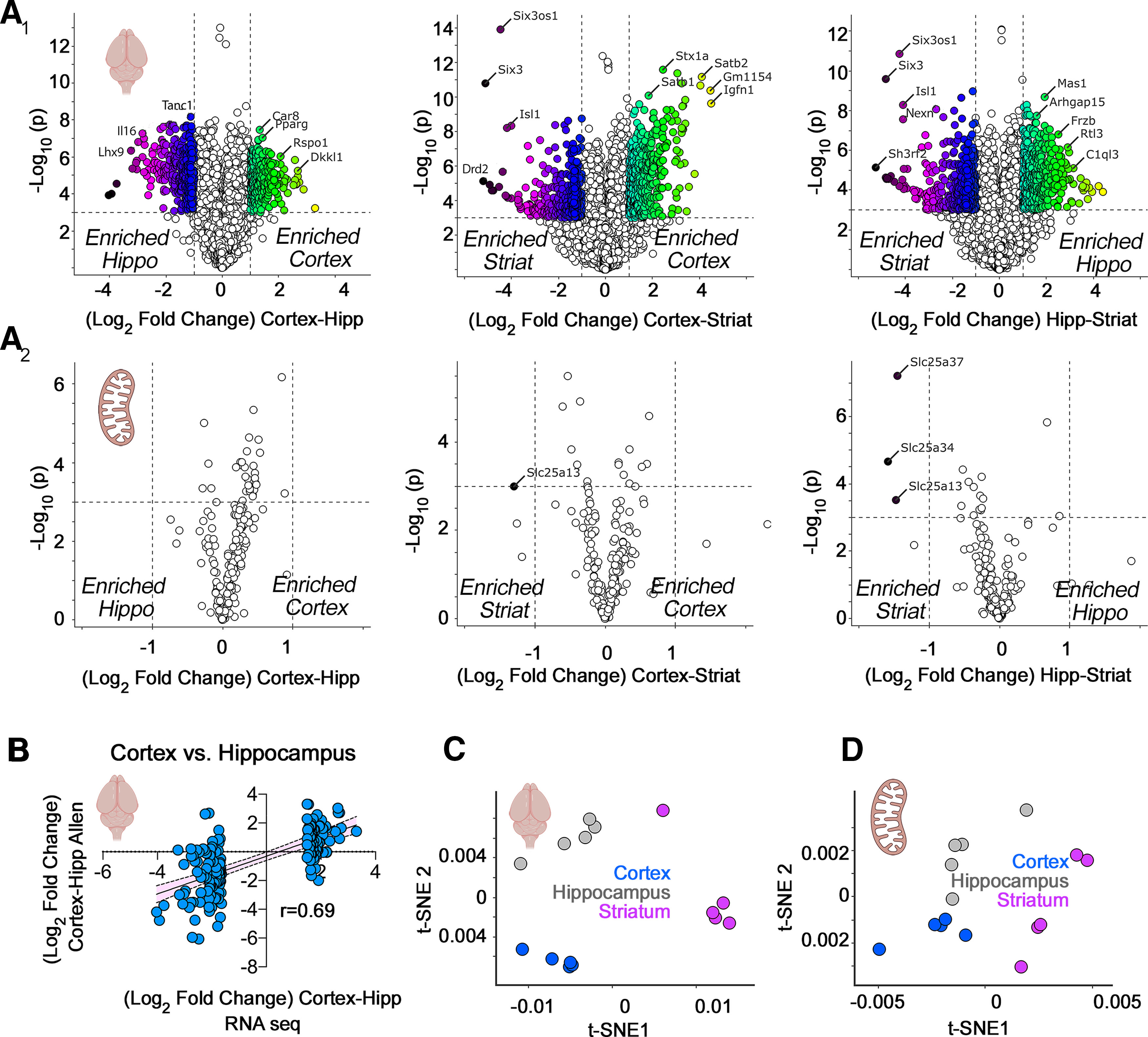
RNAseq analysis of microdissected mouse brain regions. ***A_1_***, ***A_2_***, Volcano plots of cortex compared with hippocampus, cortex compared with striatum, and hippocampus compared with striatum from adult male mice (*n* = 5). Threshold for significance was set at *p* < 10^−3^ and log_2_ fold change at 1. Color code symbols depict the fold of change below or above the thresholds. ***A_1_***, All transcripts quantified using DSeq2 annotated to the mouse genome GRCm38. ***A_2_***, All nuclear transcripts encoding subunits of the respiratory chain complexes, the mitochondrial ribosome, and the SLC25A family of transporters. Note that scarce numbers of these nuclear encoded mitochondrial transcripts show modest expression differences among brain regions. ***B***, Validation of the RNAseq results using as a comparison the *in situ* hybridization data from the Allen Mouse Brain Atlas. The 100 most upregulated and downregulated genes when comparing cortex and hippocampus by RNAseq were correlated with the differences reported by the Allen data. ***C***, t-SNE analysis of the RNAseq data presented in ***A_1_***. ***D***, t-SNE analysis of the data presented in ***A_2_***.

We compared the top 100 genes whose expression was different between cortex and hippocampus data from our RNAseq study to the quantitative *in situ* hybridization data from the mouse brain atlas at the Allen Institute. Correlation analysis was performed with Prism 9 for macOS version 9.1.1 (223), see Fig. 1.

### MS

#### Sample processing

Each tissue piece was individually homogenized in 500 μl of urea lysis buffer (8 m urea and 100 mm NaHPO_4_, pH 8.5), including 5 μl (100× stock) HALT protease and phosphatase inhibitor cocktail (Pierce). All homogenization was performed using a Bullet Blender (Next Advance) according to manufacturer protocols. Briefly, each tissue piece was added to urea lysis buffer in a 1.5 ml Rino tube (Next Advance) harboring 750-mg stainless steel beads (0.9–2 mm in diameter) and blended twice for 5-min intervals at 4°C. Protein supernatants were transferred to 1.5-ml Eppendorf tubes and sonicated (Sonic Dismembrator, Fisher Scientific) three times for 5 s with 15-s intervals of rest at 30% amplitude to disrupt nucleic acids and subsequently vortexed. Protein concentration was determined by the bicinchoninic acid (BCA) method, and samples were frozen in aliquots at −80°C. Protein homogenates (100 μg) were diluted with 50 mm NH_4_HCO_3_ to a final concentration of less than 2 m urea and then treated with 1 mm dithiothreitol (DTT) at 25°C for 30 min, followed by 5 mm iodoacetimide (IAA) at 25°C for 30 min in the dark. Protein was digested with 1:100 (w/w) lysyl endopeptidase (Wako) at 25°C for 2 h and further digested overnight with 1:50 (w/w) trypsin (Promega) at 25°C. Resulting peptides were desalted with a Sep-Pak C18 column (Waters) and dried under vacuum.

#### Tandem mass tag (TMT) labeling

For each tissue type, 10 individual samples and one composite sample were labeled using the TMT 11-plex kit (ThermoFisher 90406). Labeling was performed as previously described (Ping et al., 2018; Higginbotham et al., 2020). Briefly, each sample containing 100 μg of peptides was re-suspended in 100 mm TEAB buffer (100 μl). The TMT labeling reagents were equilibrated to room temperature, and anhydrous ACN (256 μl) was added to each reagent channel. Each channel was gently vortexed for 5 min, and then 41 μl from each TMT channel was transferred to the peptide solutions and allowed to incubate for 1 h at room temperature. The reaction was quenched with 5% (v/v) hydroxylamine (8 μl; Pierce). All 10 channels were then combined and dried by SpeedVac (LabConco) to ∼150 μl and diluted with 1 ml of 0.1% (v/v) TFA, then acidified to a final concentration of 1% (v/v) FA and 0.1% (v/v) TFA. Peptides were desalted with a 200 mg C18 Sep-Pak column (Waters). Each Sep-Pak column was activated with 3 ml of methanol, washed with 3 ml of 50% (v/v) ACN, and equilibrated with 2 × 3 ml of 0.1% TFA. The samples were then loaded and each column was washed with 2 × 3 ml 0.1% (v/v) TFA, followed by 2 ml of 1% (v/v) FA. Elution was performed with 2 volumes of 1.5 ml 50% (v/v) ACN. The eluates were then dried to completeness.

#### High pH fractionation

High pH fractionation was performed essentially as described with slight modification (Ping et al., 2020). Dried samples were re-suspended in high pH loading buffer (0.07% v/v NH_4_OH, 0.045% v/v FA, 2% v/v ACN) and loaded onto an Agilent ZORBAX 300 Extend-C18 column (2.1 × 150 mm with 3.5-μm beads). An Agilent 1100 HPLC system was used to carry out the fractionation. Solvent A consisted of 0.0175% (v/v) NH_4_OH, 0.0125% (v/v) FA, and 2% (v/v) ACN; solvent B consisted of 0.0175% (v/v) NH_4_OH, 0.0125% (v/v) FA, and 90% (v/v) ACN. The sample elution was performed over a 58.6-min gradient with a flow rate of 0.4 ml/min. The gradient consisted of 100% solvent A for 2 min, then 0–12% solvent B over 6 min, then 12–40% over 28 min, then 40–44% over 4 min, then 44–60% over 5 min, and then held constant at 60% solvent B for 13.6 min. A total of 96 individual equal volume fractions were collected across the gradient and subsequently pooled by concatenation into 24 fractions and dried to completeness using a vacuum centrifugation.

#### Liquid chromatography tandem MS

Each of the 24 high-pH peptide fractions was resuspended in loading buffer (0.1% FA, 0.03% TFA, 1% ACN). Peptide eluents were separated on a self-packed C18 (1.9 μm Maisch) fused silica column [25 cm × 75 μm internal diameter (ID), New Objective] by an Easy nLC 1200 (Thermo Scientific) and monitored on an Q-Exactive HFX MS (Thermo Scientific). Elution was performed over a 120 min gradient at a rate of 300 nl/min with buffer B ranging from 3% to 40% (buffer A: 0.1% FA in water; buffer B: 0.1% FA in 80% ACN). The mass spectrometer was set to acquire data in positive ion mode using data-dependent acquisition with top 10 cycles. Each cycle consisted of one full MS scan followed by a maximum of 10 MS/MS. Full MS scans were collected at a resolution of 120,000 (400–1600 m/z range, 3 × 10̂6 AGC, 100 ms maximum ion injection time). All higher energy collision-induced dissociation (HCD) MS/MS spectra were acquired at a resolution of 45,000 (1.6 m/z isolation width, 30% collision energy, 1 × 10^–5^ AGC target, 86-ms maximum ion time). Dynamic exclusion was set to exclude previously sequenced peaks for 20 s within a 10-ppm isolation window.

#### Data processing protocol

All raw files were searched using Thermo’s Proteome Discoverer suite (version 2.1.1.21) with Sequest HT. The spectra were searched against a mouse Uniprot database downloaded July, 2018 (98,225 target sequences). Search parameters included 20-ppm precursor mass window, 0.05-Da product mass window, dynamic modifications methione (+15.995 Da), deamidated asparagine and glutamine (+0.984 Da), phosphorylated serine, threonine and tyrosine (+79.966 Da), and static modifications for carbamidomethyl cysteines (+57.021 Da) and N-terminal and lysine-tagged TMT (+229.26340 Da). Percolator was used filter PSMs to 0.1%. Peptides were grouped using strict parsimony and only razor and unique peptides were used for protein level quantitation. Reporter ions were quantified from MS2 scans using an integration tolerance of 20 ppm with the most confident centroid setting. Only unique and razor (i.e., parsimonious) peptides were considered for quantification.

The MS proteomics data have been deposited to the ProteomeXchange Consortium via the PRIDE (Perez-Riverol et al., 2019) partner repository with the dataset identifier PXD026104.

**Figure 2.**
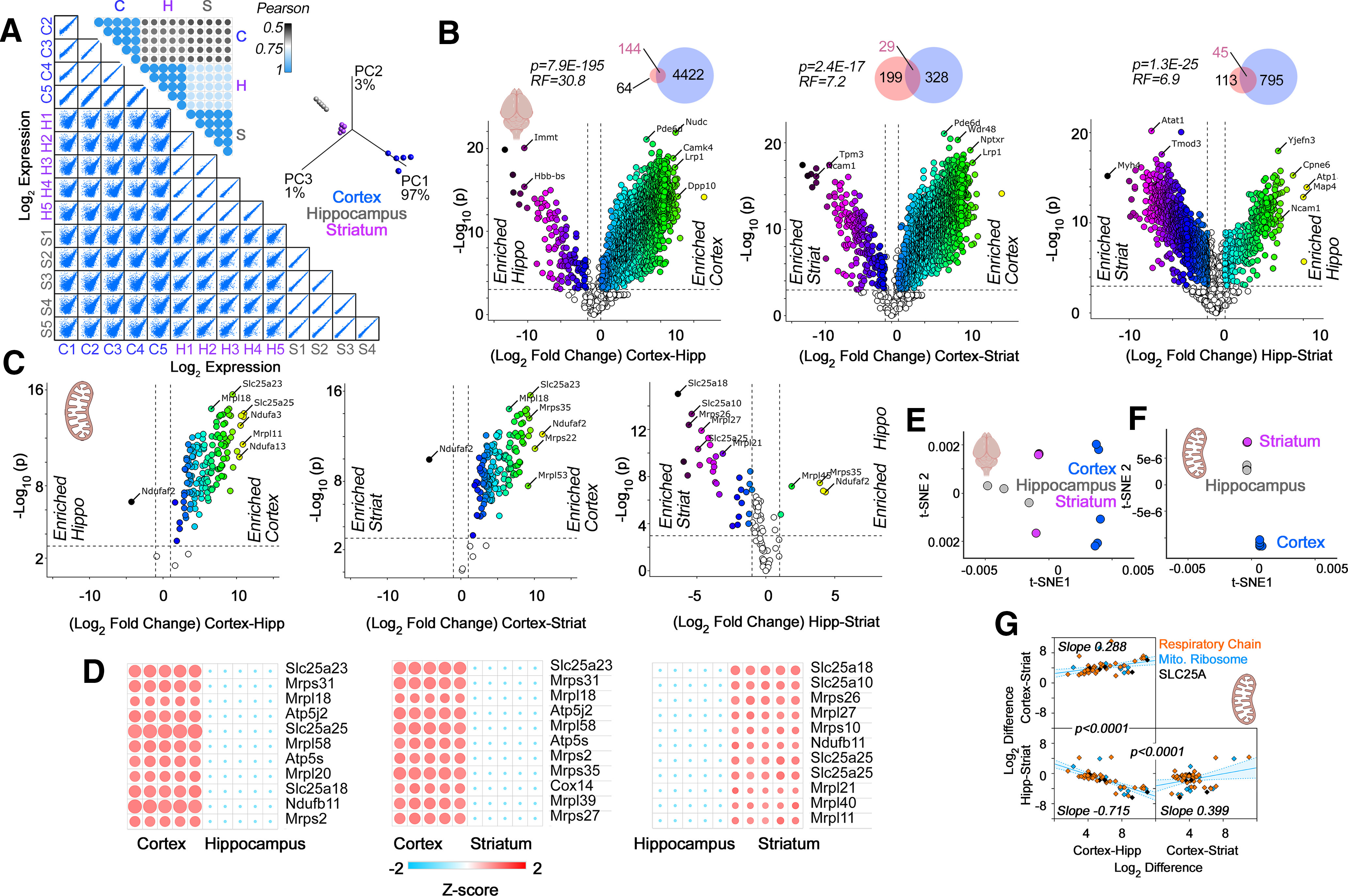
TMT proteomic analysis of microdissected mouse brain regions. ***A***, Multiscatter plots with all individual biological replicates used for TMT quantifications. Insets, Pearson similarity coefficients and PCA of samples in multiscatter plots. ***B***, ***C***, Volcano plots of cortex compared with hippocampus, cortex compared with striatum, and hippocampus compared with striatum from adult male mice (*n* = 5). Threshold for significance were set at *p* < 10^−3^ and log_2_ fold change at 1. Color code symbols depict the fold of change below or above the thresholds. ***B***, All proteins quantified in brain samples with inset Venn diagrams depicting the overlap between our TMT data (blue) and label-free quantifications by Sharma et al. (2015; pink). Representation factor and *p* values were estimated with an exact hypergeometric probability test. ***C***, All nuclear encoded subunits of the respiratory chain complexes, the mitochondrial ribosome, and the SLC25A family of transporters. Note the abundant nuclear encoded mitochondrial proteins differentiating brain regions. ***D***, Heat maps of the proteins that show the most pronounced changes based on the *q* value and magnitude of the difference. ***E***, t-SNE analysis of the proteome data presented in ***B***. ***F***, t-SNE analysis of the data presented in ***C***. Note that the best clustering of brain regions is obtained with the nuclear encoded mitochondrial proteins described in ***C***. ***G***, Simple linear correlation analysis of expression differences across brain regions. Proteins belonging to respiratory chain complexes, the mitochondrial ribosome, and the SLC25A family of transporters are color coded. Note the differences in slopes. p values describe the differences between adjacent correlation plots slopes obtained with Prism. Shaded area represents the 95% confidence interval. See Extended Data Figure 2-1 for list of protein hits with *p* < 10^−3^ and log_2_ fold change of least 1.

Correlations and statistical analysis between the fold of change expression slopes of the selected 210 nuclear encoded mitochondrial transcripts presented in Figure 2*G* was performed with Prism 9 for macOS version 9.1.1 (223).

10.1523/ENEURO.0232-21.2021.f2-1 Extended Data Figure 2-1 Significant TMT MS hits curated by region and mitochondrial localization. Download Figure 2-1, XLSX file.

### Single-cell RNAseq

Single-cell RNAseq data are described in (Yao et al., 2020). Gene expression data matrix (matrix.csv) and cell metadata (metadata.csv). Whole cortex and hippocampus-smart-seq (2019) with 10×-smart-seq taxonomy (2020) data were downloaded from the Allen Institute Portal. This dataset contains RNAseq data of single cells isolated from >20 areas of mouse cortex and hippocampus. Abbreviations used in figures match the Allen Mouse Brain Atlas. The data set includes 76,307 single cells. The sequencing results were aligned to exons and introns in the GRCm38.p3 reference genome using the STAR algorithm, and aggregated intron and exon counts at the gene level were calculated.

Matrix files were processed with Delimit Pro for Windows 10/8.1/7. We selected the 210 nuclear encoded transcripts from the matrix.csv file with Delimit Pro and data were assembled in Excel together with the metadata.csv data. Data were exported as tab delimited text file and analyzed with the Qlucore Omics Explorer version 3.6(33). Data were log2 converted and normalized to a mean of 0 and a variance of 1. 2D t-distributed stochastic neighbor embedding (t-SNE) plots were generated using a perplexity of 10 and default settings. Callouts were made by cell metadata or gene expression levels. Respiratory complexes and mitochondrial ribosome subunits were defined using the CORUM database, see text for complex entries (Giurgiu et al., 2019).

## Results

### Brain expression of mitochondrial proteins reveals regional heterogeneity

Expression levels of proteins and their transcripts have been used to explore tissue heterogeneities in organelle abundance and/or composition (Andersen and Mann, 2006; Geiger et al., 2013; Wilhelm et al., 2014; Cardoso-Moreira et al., 2019; He et al., 2020). We applied this paradigm to mitochondria from adult mouse brain regions. We performed simultaneous quantifications of the transcriptome and proteome from punch-microdissected mouse coronal sections of cortex, hippocampus, and the striatum. We chose punch-microdissected tissue to minimize noise introduced by tissue heterogeneity. Microdissection resulted in tissue samples of ∼160 μm^3^ for microanalytical omics analyses. We focused on components of the five electron transport chain complexes, the mitochondrial ribosome, and the SLC25A family of inner mitochondrial membrane transporters. We selected the five respiratory chain complexes and the mitochondrial ribosome, as these complexes are necessary components of mitochondria and have defined subunit stoichiometries necessary for their function (Vafai and Mootha, 2012). In contrast, the expression of SLC25A transporter family members is variable among tissues, as only the phosphate carrier (SLC25A3) and ADP/ATP carriers (SLC25A4-6) are essential for ATP synthesis (Cunningham and Rutter, 2020; Palmieri et al., 2020; Rath et al., 2021). Collectively, this set of genes constituted 210 proteins, or 18% of the mitochondria-annotated proteome (Rath et al., 2021). We reasoned that respiratory chain complex subunits and the mitochondrial ribosome should be refractory to anatomic expression differences because of their fixed stoichiometries, while the SLC25A family of transporters would be likely to reveal heterogeneous expression across brain regions.

**Figure 3.**
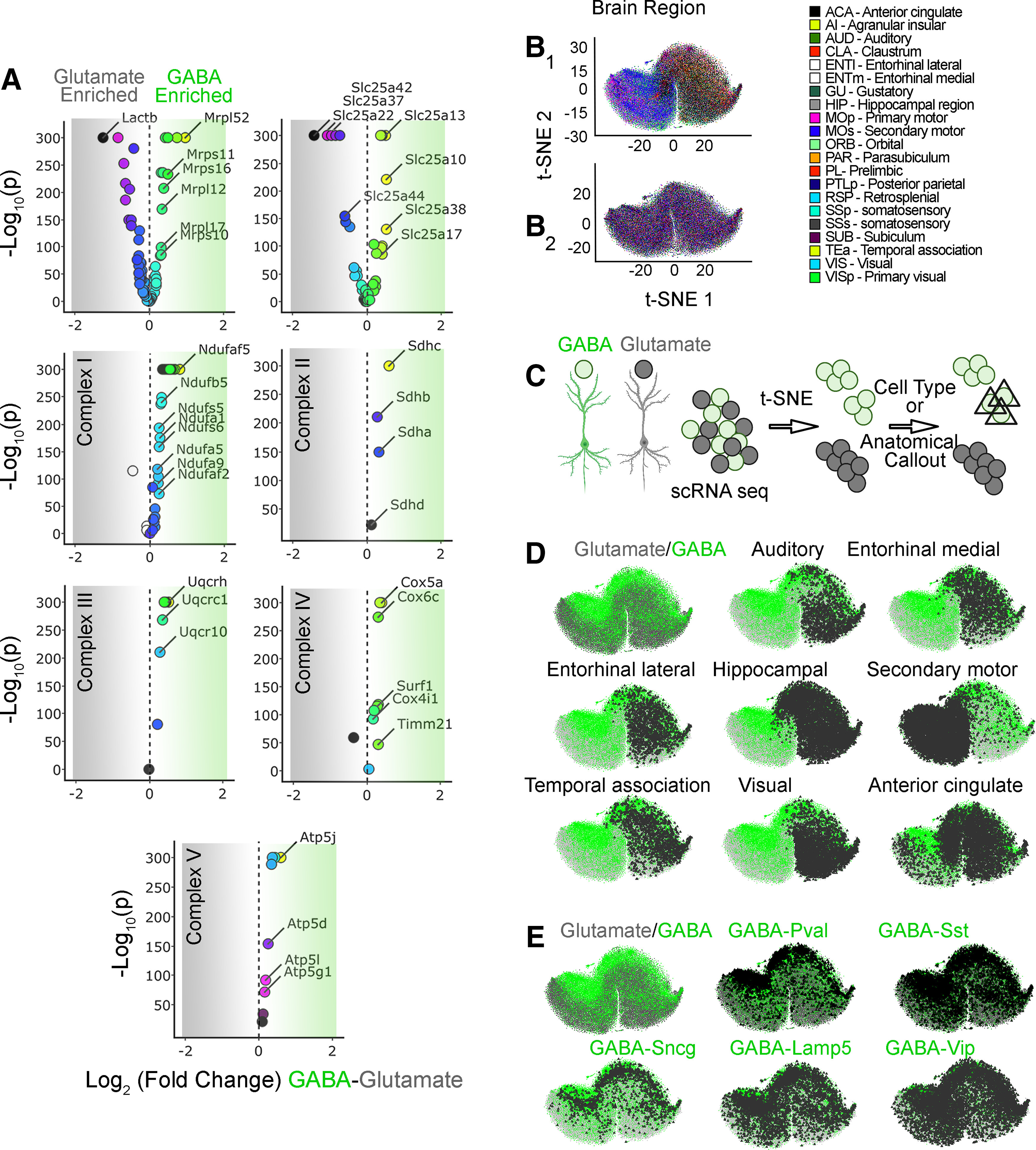
Nuclear encoded mitochondrial transcripts differentiate neurons by neurotransmitter identity and anatomical location. ***A***, Volcano plots were assembled using the Allen single-cell RNAseq dataset. A total of 50,002 pyramidal glutamatergic neurons were compared with 22,745 GABAergic interneurons. Volcano plots are organized by subunits belonging to the mitochondrial ribosome, electron chain complexes I to V, and the SLC25A family of solute transporters. The mitochondrial ribosome and the SLC25A family of transporters are the most dissimilarly expressed transcripts when comparing GABAergic with glutamatergic neurons. ***B_1_***, t-SNE cell atlas generated with the expression levels of all transcripts encoding mitochondrial ribosome subunits. The t-SNE atlas encompasses >20 areas of mouse cortex and hippocampus, totaling 76,307 cells. Color codes denote brain regions annotated by the Allen Brain Atlas. ***B_2_*** shows ***B_1_*** data after 100 consecutive permutations. Anatomical segregation is lost. ***C***, Diagram explaining strategy for cell type and anatomic callout in t-SNE atlases. GABAergic neurons were color-coded green and glutamatergic neurons were color coded gray. Cell type and anatomic region were marked by a triangle. ***D***, t-SNE atlas shown in ***B_1_*** that was layered with the neurotransmitter identity of cells and anatomic location of cells (triangles). GABA, parvalbumin (Pval), somatostatin (Sst), γ-synuclein (Sncg), vasointestinal peptide (Vip), and lysosomal-associated membrane protein family member 5 (Lamp5) denote markers defining specific interneuron subpopulations. ***E***, t-SNE atlas shown in ***B_1_*** but layered with the subtype of interneuron (triangles).

We quantified mRNA expression across three distinct mouse brain regions encompassing diverse cell types: cortex, hippocampus, and striatum (Fig. 1). We focused first on all mRNAs encoded in the mouse genome (Fig. 1*A1*), and then on a subset of 210 of these messages encoding proteins localized to mitochondria (Fig. 1*A2*). We considered an expression change significant if gene expression between two regions differed by at least 2-fold with *p* < 0.001. These same thresholds were applied to RNAseq and proteome datasets from mouse tissues (Figs. 1-3).

Brain regions were discriminated by their whole-genome transcript expression (Fig. 1*A1*). For example, cortex and hippocampus differed by 353 genes whose relative expression was higher in cortex and 316 genes whose relative expression was higher in hippocampus (Fig. 1*A1*). We validated these gene expression differences with the Allen Mouse Expression Atlas and observed a strong correlation between both datasets (*r* = 0.69, *p* < 0.0001; Fig. 1*B*). In contrast with the transcriptomes of whole brain regions, the 210 mRNAs mapping to the selected subset of nuclear encoded mitochondrial proteins have minimal expression differences among the three brain regions (Fig. 1*A2*). We could only distinguish the striatum from other regions because of its low expression of the transporters SLC25A13, SLC25A34, and SLC25A37 (Fig. 1*A2*). These transporters encode an aspartate-glutamate exchanger, an orphan transporter, and an iron uptake transporter, respectively (Palmieri and Monné, 2016). While global gene expression patterns segregated cortex and other brain regions into defined clusters by t-SNE (Fig. 1*C*; Kobak and Berens, 2019), the 210 selected mitochondrial transcripts poorly distinguished brain regions using t-SNE analysis (Fig. 1*D*). This indicated minimal regional differences in the bulk expression of messages encoding proteins localized to mitochondria.

**Figure 4.**
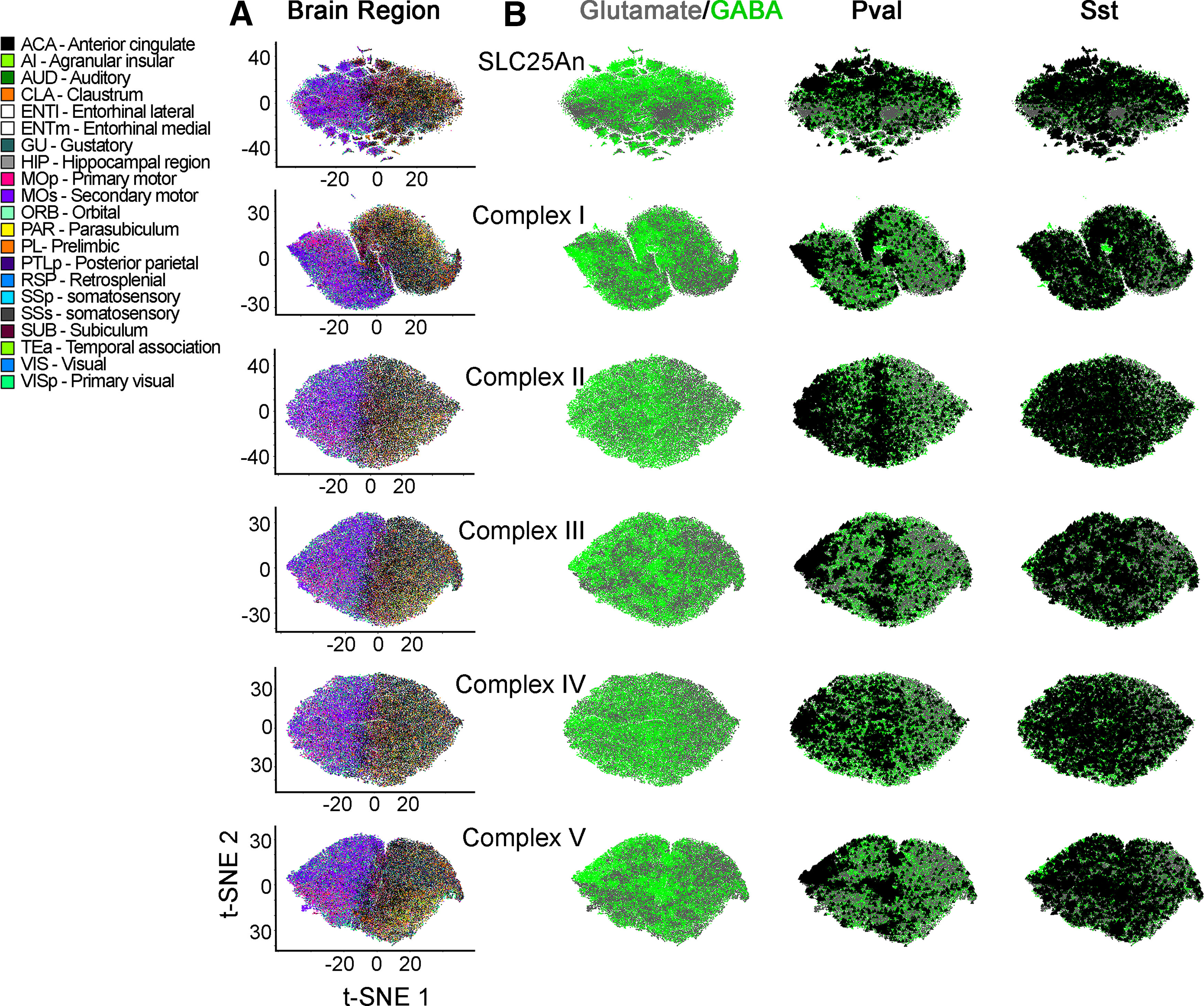
Families of nuclear encoded mitochondrial transcripts differentiate neurons by neurotransmitter identity and anatomical location. ***A***, ***B***, t-SNE cell atlases were generated with the expression levels of nuclear transcripts either encoding subunits of the respiratory chain complexes I to V, the mitochondrial ribosome, or the SLC25A family of transporters. ***A***, t-SNE atlases encompasses >20 areas of mouse cortex and hippocampus, totaling 76,307 cells in each case. Color codes denote brain regions annotated by the Allen Brain Atlas. ***B***, GABAergic neurons color-coded green and glutamatergic neurons color coded gray. PV-positive and somatostatin-positive cells were marked by a triangle. Note that families of transcripts can segregate cells by their lineage and anatomic origin.

**Figure 5.**
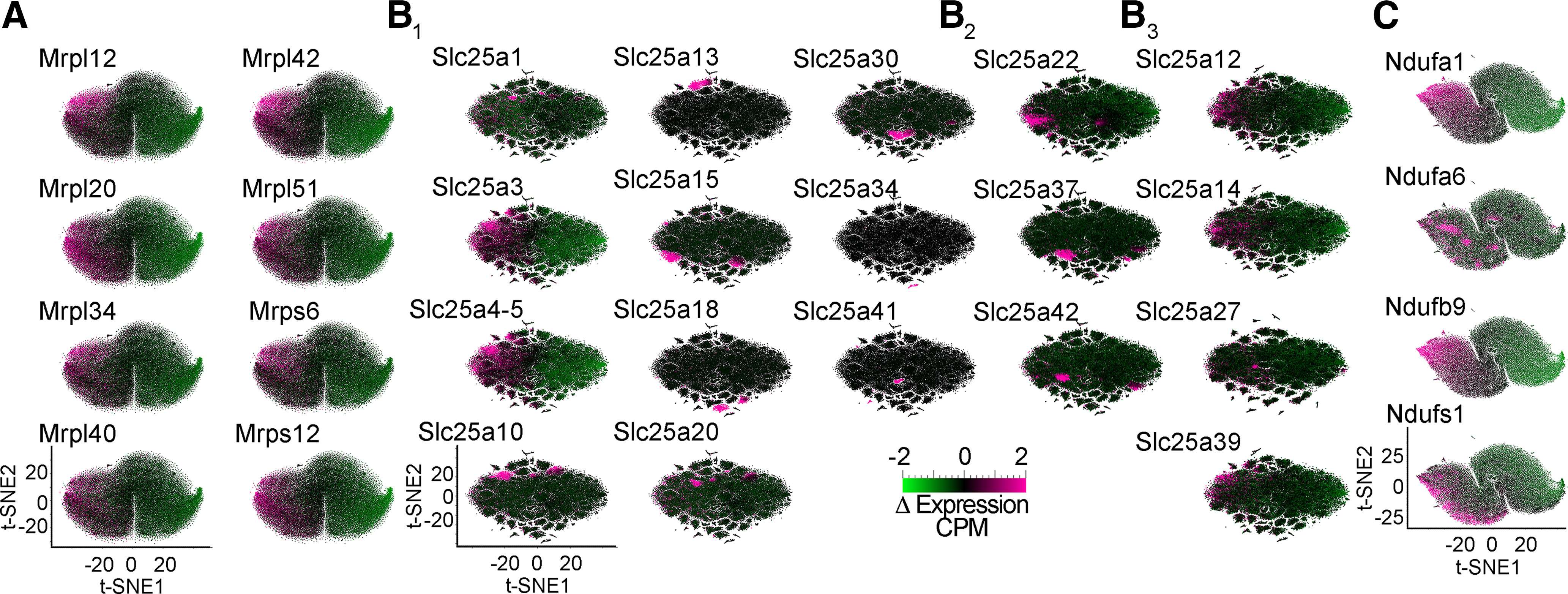
Differential expression of selected nuclear encoded mitochondrial transcripts further differentiates neuronal subpopulations. ***A–C***, t-SNE cell atlases built with the subunits of the mitochondrial ribosome (***A***), the SLC25A family of transporters (***B***), and the electron transport chain Complex I (***C***). t-SNE cell atlases were overlaid with heat maps of the expression levels of selected subunits of protein complexes or transporters. ***B_1_***, Transporters diffusely expressed across brain regions or showing specific patterns of expression. ***B_2_***, Transporters Slc25a22, Slc25a37, and Slc25a42 preferentially expressed in glutamatergic cells (see Fig. 3*A*). ***B_3_***, SLC25A transporters annotated in the SFARI database associated with autism spectrum disorder (SLC25A12, SLC25A27, and SLC25A39) or whose expression is altered in postmortem autism brain samples (SLC25A12, SLC25A14, and SLC25A27).

The poor discrimination between brain regions by the 210 mRNAs encoding our selected subset of mitochondrial proteins could be interpreted in the following ways. Anatomical differences in cellular composition could skew regional differences dictated by these mRNAs. For instance, increased numbers of mitochondria in certain cell types may mask any differences that would otherwise be detectable when analyzing single cells. Additionally, regional differences could be manifested at the protein rather than at the transcript level. This last problem is a common occurrence in diverse tissues and cell types, including the brain, with correlations between mRNA and protein expression below 0.5 (de Sousa Abreu et al., 2009; Ghazalpour et al., 2011; Schwanhäusser et al., 2011; Carlyle et al., 2017; Wang et al., 2019). We addressed these questions by quantifying regional proteomes in mouse brain (Fig. 2) and by analyzing the expression of these 210 mitochondrial transcripts at a single-cell level (Figs. 3-5). We used quantitative isobaric labeling by TMT of adult mouse brain proteomes to measure regional proteome differences (Fig. 2; Werner et al., 2012; Gokhale et al., 2019, 2021). We selected TMT MS quantification of the proteome because TMT offers improved capacity to detect changes reaching statistical significance. This is because of TMT’s superior precision and reduced number of missing values as compared with label-free quantifications (O’Connell et al., 2018).

We first determined the quality of our 15-plex TMT brain proteome across the three brain regions selected, analyzing correlation coefficients between biological replicates within and in between brain regions (Fig. 2). Multiscatter plots and correlation matrices showed Pearson correlations >0.9 among biological replicates within a brain region and above 0.5 in comparisons between regions (Fig. 2*A*). These strong correlations manifested as a reduced variance by principal component analysis (PCA) where 97% of the total variance was accounted by principal component 1 (Fig. 2*A*, inset). Biological replicates within regions clustered closely and were segregated from other brain regions by PCA, thus validating our TMT proteome dataset (Fig. 2*A*, inset).

The global proteome unveiled vast differences between mouse brain regions (Fig. 2*B*). These proteome differences were more pronounced than those found at the transcript level. As an example, the cortex and hippocampus differed in the expression levels of 4698 proteins among a total of 5285 proteins quantified by TMT, while these two regions differed significantly in only 670 mRNAs (compare Figs. 1*A1* and 2*B*). We further validated our findings and datasets by comparing our proteome hits against the label-free quantified proteomes by Sharma et al. (2015). Our mouse cortex-enriched proteome captured 69.2% of the mouse motor cortex-enriched proteome described by Sharma et al. (2015). This overlap is 30.8 times above what it is expected by chance (*p* = 7.9E-195; Fig. 2*B*, Venn diagram insets). We also found significant, yet less pronounced, overlaps between the hippocampal-enriched and striatum-enriched hits and those previously reported (Sharma et al., 2015). Thus, our results capture previously reported differences in the regional brain proteomes and significantly expand them by deploying TMT MS as a way to quantify the proteome.

Among proteins whose expression differed across brain regions, we found multiple mitochondrial proteins (Fig. 2*C*,*D*). The most significant changes in mitochondrial protein expression included proteins belonging to respiratory chain complexes, the mitochondrial ribosome, and the SLC25A family of transporters (Fig. 2*C*,*D*). The highest expression levels of some of these mitochondrial proteins were observed in the cortex (Fig. 2*C*). We used a nonlinear tool of data dimensionality reduction, t-SNE, to uncover similarities in the local and global structure of the protein expression data (Fig. 2*E*,*F*; Kobak and Berens, 2019). t-SNE analysis of the whole proteome showed that the three brain regions studied did not group into clearly defined clusters (Fig. 2*E*). However, when t-SNE analysis was performed with the selected mitochondrial proteins, cortex, hippocampus, and striatum were group into clearly distinct cluster (Fig. 2*F*). Thus, t-SNE analysis indicates that expression differences in mitochondrial proteins alone can anatomically discriminate these datasets.

To further explore the regional differences we observed in mitochondrial protein expression, we performed correlation analysis of these differences across paired brain regions (Fig. 2*G*), focusing on the selected mitochondrial proteins of interest. We reasoned that anatomically universal mitochondrial expression patterns would be represented by similar expression differences across multiple mitochondrial proteins between two regions. Similar slopes among regional pairwise comparisons would indicate homogenous expression differences, while differences in slope would suggest regional composition differences. These compositional distinctions could originate either from differences in mitochondria shared by all cells in a defined anatomic location or differences in mitochondrial composition among diverse cell types residing in a defined anatomic region (Fig. 2*G*). We found that pairwise expression difference correlations showed different ordinate intersects (Fig. 2*G*). The slope of these correlations was significantly distinct among regional pairwise comparisons (Fig. 2*G*). Moreover, subunits of respiratory chain complexes and the mitochondrial ribosome were similarly weighted to the parameters of these correlations (Fig. 2*G*, orange and blue symbols, respectively). These data argue for regional heterogeneity in the expression of mitochondrial constituents, even among respiratory chain complexes and the mitochondrial ribosome.

### Single-cell transcriptomes identify anatomic and cell type-specific differences in nuclear encoded mitochondrial genes

The proteomics data suggested that regional heterogeneities in mitochondrial protein expression in adult mouse brain could originate from intrinsic differences in the cellular expression of nuclear encoded mitochondrial genes. To test this hypothesis, we analyzed the expression of the 210 nuclear encoded mitochondrial transcripts at the single-cell level. We reasoned the ineffectiveness of bulk tissue RNAseq discriminating brain regions solely on nuclear encoded mitochondrial transcripts (Fig. 1) could be bypassed by the richness of fine-grained categorizational information from single-cell RNAseq datasets. We selected the Allen single-cell transcript expression dataset as the most comprehensive single-cell transcript expression study to date (Yao et al., 2020). The Allen brain dataset encompasses >20 areas of mouse cortex and hippocampus, totaling 76,307 cells. Of these cells, 50,002 correspond to pyramidal glutamatergic neurons and 22,745 correspond to GABAergic interneurons, which include 4363 parvalbumin (PV)-positive cells (Yao et al., 2020).

We asked whether the mRNA expression of any one of the five electron transport chain complexes, the mitochondrial ribosome, or the SLC25A family of inner mitochondrial membrane transporters was able to discriminate anatomic regions and brain cell types in t-SNE-generated atlases (Figs. 3-5). We first sought to determine whether the expression of transcripts could be different between glutamatergic and GABAergic neurons when single-cell expression was bulk averaged across each cell category. Volcano plots revealed that the most pronounced changes in the number of transcripts and the magnitude of expression differences occurred among subunits of the mitochondrial ribosome and the SLC25A family of mitochondrial transporters (Fig. 3*A*). Some transcripts were enriched in glutamatergic neurons, such as the mitochondrial ribosome subunit Lactb (Mrpl56) or the mitochondrial glutamate/proton symporter SLC25A22 (Fig. 3*A*). Conversely, the mitochondrial ribosome subunit Mrpl52 and the mitochondrial aspartate-glutamate carrier SLC25A13 were enriched in GABAergic neurons (Fig. 3*A*). The bulk expression of at least one respiratory complex subunit or its assembly factor was substantially different between these two neuron types (Fig. 3*A*; see Ndufaf5, Sdhc, and Atp5J).

We used t-SNE to compress multidimensional mRNA expression data into single point cell representations. t-SNE atlases capture and represent similarities in single-cell gene expression by clustering cells along the coordinates of a bidimensional space (Fig. 3*B*; Kobak and Berens, 2019). These atlases were then annotated based on their anatomic location or cell type, using triangles to label cells belonging to a particular region (Fig. 3*C–E*). We focused on GABAergic (Fig. 3*C–E*, green dots) and glutamatergic neurons (Fig. 3*C–E*, gray dots), as these two cell types were the most numerous cells whose gene expression was scored in the Allen dataset (Yao et al., 2020).

We next focused our analysis on the mitochondrial ribosome, as this organelle is the biggest protein complex in mitochondria. The ribosome is encoded by ∼80 core nuclear expressed proteins necessary for organelle function (CORUM complex #320; Giurgiu et al., 2019). We built a mitochondrial ribosome subunit expression t-SNE atlas (Fig. 3*B1*). This atlas revealed that mitochondrial ribosome mRNA expression profiles grouped cells into distinct areas of the cortex and hippocampus (the hippocampus is annotated by color in Fig. 3*B1* and by a triangle callout in Fig. 3*D*), as well as neuronal cell types within these regions (Fig. 3*D*, anatomic annotation by triangle callout over a color-coded glutamate vs GABA-annotated atlas). These anatomic distinctions were removed after data permutation, supporting specific anatomic patterns of mitochondrial ribosome gene expression across the brain (Fig. 3*B2*). t-SNE analysis of mitochondrial ribosome gene expression segregated cells into two major clusters; one was enriched in cells from the temporal cortex, visual cortex, and hippocampus, while the other cluster preferentially enriched cells from motor cortex areas (Fig. 3*D*, anatomic annotation by triangle callout over a color-coded glutamate vs GABA-annotated atlas). We mapped the neurotransmitter identity of different cell types into this mitochondrial ribosome gene expression atlas to assess cell type-specific variations in gene expression (Fig. 3*E*, GABAergic neuronal subtype annotation by triangle callout over a color-coded glutamate vs GABA-annotated atlas). t-SNE analysis revealed clear distinctions between GABAergic and glutamatergic cells (Fig. 3*E*, green and gray symbols, respectively). In particular, PV-positive and γ-synuclein (Sncg)-positive interneurons were the most clearly segregated cell types, regardless of their anatomic location (compare triangle callouts in Fig. 3*D*,*E*). Cell clustering was less pronounced for somatostatin-positive or vasointestinal peptide-positive interneurons (Fig. 3*E*). We overlapped the t-SNE transcriptional clusters with heat maps depicting expression levels of representative mitochondrial ribosome transcripts (Fig. 5). These heat maps indicated that the expression of several mitochondrial ribosome transcripts was higher in GABAergic interneurons, in particular PV-positive interneurons localized to motor areas of the cortex (compare Figs. 3*D*,*E* and 5*A*).

We wanted to evaluate the robustness of nuclear encoded mitochondrial gene expression sets to segregate cell populations into anatomic and cell type categories (Fig. 4). To this end, we built additional gene expression atlases with transcript datasets made up of either the 48 SLC25A transporters, 45 subunits of Complex I, four subunits of Complex II (CORUM complex #440), 10 subunits of Complex III (CORUM complex #403), 14 subunits of Complex IV (CORUM complex #6442), or 16 subunits of Complex V (CORUM complex #563; Giurgiu et al., 2019). Each one of these atlases segregated cells into distinct anatomic and cell type-specific cell populations (compare Fig. 4*A*, where anatomic annotation is done by color, and *B*, where the GABAergic cell type annotation is done with triangle callouts). Of note, the distance to nearest neighbors for the SLC25A family was more variable, containing small distinct clusters, compared with the ribosome and electron transport chain complexes. Transcriptionally defined cell populations were identified regardless of the complexity of the dataset fed into the t-SNE algorithm. For example, t-SNE analysis of Complex II, a complex represented just by four transcripts, segregated cells into clusters categorized by anatomic location and cell type (Fig. 4*A*,*B*). The expression of Complex II subunits was sufficient to distinguish PV-positive GABAergic neurons among all cell types, regardless of their anatomic location (Fig. 4*A*,*B*). Similar findings were obtained with cell atlases generated with each one of the respiratory chain complexes, as well as the SLC25A transporter family (Fig. 4*A*,*B*).

We also determined whether the expression levels of different transcripts could further distinguish transcriptionally defined cell populations. We superimposed expression heat maps of mitochondrial ribosome subunits, transporters of the SLC25A family, and subunits of Complex I into their corresponding atlases. Subunits of the mitochondrial ribosome expressed similarly and preponderantly in GABAergic neurons (compare Figs. 5*A* and 3*E*); however, members of the SLC25A transporter family displayed variable transcript expression patterns (Fig. 5*B*). On one hand, we found SLC25A transporters whose expression was higher in classes of GABAergic neurons (compare Figs. 5*B1* and 4*A*). These include the phosphate transporter SLC25A3 and the ADP-ATP mitochondrial translocators SLC25A4 and SLC25A5. These three SLC25A transporters are indispensable in mitochondria for ATP generation (Palmieri and Monné, 2016; Cunningham and Rutter, 2020). On the other hand, we identified transporters whose expression was 2-fold to 4-fold higher in discrete cell populations. For example, SLC25A13 was expressed at high levels in a unique subgroup of cells among PV-positive cells (compare Fig. 5*B1* and 4*A*). This cell population is missed in the expression analysis of this transporter when glutamatergic and GABAergic neuron SLC25A13 mRNA levels were averaged in bulk (Fig. 3*A*, volcano plots). Similarly, the most expressed transporters in glutamatergic cells by bulk averaging were SLC25A22, SLC25A37, and SLC25A42 (Fig. 3*A*). Yet, when we mapped the expression levels of these transporters to a t-SNE atlas, we found again discrete cell populations with uniquely high expression levels of these three transporters (Fig. 5*B2*).

We expanded our studies to the expression of a subset of SLC25A transporters annotated in the SFARI database that are associated with autism spectrum disorder (SLC25A12, SLC25A27, and SLC25A39; Abrahams et al., 2013), or whose expression is altered in postmortem autism brain samples (SLC25A12, SLC25A14, and SLC25A27; Fig. 5*B3*; Segurado et al., 2005; Lepagnol-Bestel et al., 2008; Anitha et al., 2012). We found that these transporters had non-overlapping increases in expression levels in discrete brain areas and cell types (compare Figs. 5*B3* and 4*A*). Interestingly, while SLC25A12 and SLC25A13 are both aspartate-glutamate carrier isoforms, only SLC25A12 is linked to autism (Figs. 4, 5*B3*; Segurado et al., 2005; Lepagnol-Bestel et al., 2008; Palmieri and Monné, 2016; Cunningham and Rutter, 2020). The expression properties uncovered with t-SNE atlases built with mitochondrial ribosome or SLC25A transporter datasets were also evident with a Complex I subunit dataset (Fig. 5*C*). We found discrete cell clusters that differed markedly in the expression levels of some of the Complex I subunits (Fig. 5*C*, compare Ndufa1 and Ndufa6). Our findings demonstrate that PV-positive neurons and glutamatergic neurons can be differentiated based on the abundance and expression patterns of nuclear encoded mitochondrial genes. We conclude that the expression of nuclear encoded mitochondrial genes varies across anatomic locations and cell types in the brain. These findings set the stage for the possibility of diversified mitochondrial composition and function across cell types and regions in neural tissue.

## Discussion

We used proteomic and single-cell transcriptomic datasets to discern whether expression of nuclear mitochondrial genes can differentiate both anatomic regions and neuronal cell types in the adult mouse brain. We focused on a subset of nuclear mitochondrial genes encompassing the electron transport chain, mitochondrial ribosome, and SLC25A family of mitochondrial transporters. Using whole tissue datasets from the cortex, hippocampus, and striatum, we found that regional mitochondrial differences were apparent at the protein level but not the transcript level (Figs. 1, 2). We reasoned that this proteomic variation could stem from intrinsic regional differences in mitochondrial composition and/or cell type-specific mitochondrial composition, given the variable makeup of cell types in the different regions (Wheeler et al., 2015; Zeisel et al., 2015; Gokce et al., 2016; Tasic et al., 2016, 2018; Erö et al., 2018). We used t-SNE analysis of a comprehensive, neuronally-enriched single-cell RNAseq dataset to gain more insight into these possibilities (Yao et al., 2020). We found that differences in nuclear encoded mitochondrial transcript expression at the single-cell level distinguished cortical areas and regions of the hippocampal formation from one another (Figs. 2-4), and the expression of some nuclear encoded mitochondrial genes was differentially enriched in distinct cell populations in single-cell RNAseq analysis (Fig. 5). In particular, our results showed that excitatory and GABAergic neurons can be differentiated based solely on their expression of nuclear encoded mitochondrial transcripts (Fig. 3).

Our findings expand recent evidence that there is heterogeneity in mitochondrial composition among different brain cell types (Fecher et al., 2019). Fecher and colleagues used an elegant genetic approach to tag and isolate brain mitochondria in a cell type-specific manner, demonstrating that GABAergic Purkinje cells, glutamatergic granule cells, and astrocytes in the cerebellum have distinct proteomes that help carry out specialized functions in these cell types (Voogd and Glickstein, 1998; Ioannou et al., 2019). Here, we extend this evidence of heterogeneity by analyzing single-cell transcript data from a large number of neurons across diverse cortical and hippocampal areas. The breadth and granularity of our analysis extends the principle that brain mitochondria are heterogeneous organelles across diverse brain regions. We focused on electron transport chain genes, mitochondrial ribosome genes, and the SLC25A transporter family, reasoning that the defined stoichiometry of electron transport chain and ribosomal complexes would preclude them from having much heterogeneity while the SLC25A family, most of which are dispensable for ATP generation, would have more variable expression. Our proteomics data showed that, surprisingly, regional differences in mitochondrial composition extend to electron transport chain subunits and mitochondrial ribosome subunits (Fig. 2). Several mitochondrial ribosome proteins and respiratory complex proteins are enriched in the cortex compared with the hippocampus or striatum (Fig. 2). Moreover, t-SNE analysis of the single-cell RNAseq profile of mitochondrial ribosome transcript expression, SLC25A transcript expression, or expression of genes of the individual respiratory complexes segregates different cortical and hippocampal regions from one another (Figs. 3, 4). Generally, mitochondrial ribosome gene expression was enriched in GABAergic cell types, particularly fast-spiking PV-positive interneurons (compare Figs. 3*E* and 5*A*). This is perhaps not surprising given the role of the mitochondrial ribosome in translating mitochondrially encoded subunits of the electron transport chain. However, while it has been established that the mitochondrial ribosome is required for neuronal development and function (Gokhale et al., 2021), there is no evidence for GABAergic-specific requirements of mitochondrial ribosomes.

The single-cell transcriptomes of glutamatergic and GABAergic neurons, as well as different classes of GABAergic neurons, distinguish these neuronal types from one another (Fig. 3). These differences in presumed mitochondrial composition may underlie unique mitochondrial demands imposed by specialized cell types, as has been suggested by previous work (Murgia et al., 2015; Cserép et al., 2018; Fecher et al., 2019; Thomas et al., 2019). For instance, the faster spiking characteristic of PV-positive GABAergic interneurons imposes greater energy demands for these cells, and their mitochondria are ultrastructurally adapted to generate ATP very efficiently (Cserép et al., 2018). Moreover, the integrity of electron transport chain subunits is crucial for PV interneuron function (Inan et al., 2016; Sanz-Morello et al., 2020). Our data suggest that the mitochondrial ribosome and members of the SLC25A family also play key roles in PV interneuron function, as the expression of mitochondrial ribosome subunits and certain SLC25A transporters (SLC25A3-5, SLC25A13) is enriched in PV interneurons (compare Figs. 3*E*, 4, 5). GABAergic signaling by PV interneurons is key in establishing the ratio of excitatory to inhibitory (E-I) neurotransmission in the cortex (Ferguson and Gao, 2018). Disruptions to the E-I ratio have been widely hypothesized to contribute to pathogenesis of neurodevelopmental and psychiatric disorders (Nelson and Valakh, 2015; Sohal and Rubenstein, 2019). Perturbations of the E-I ratio in rodents impair circuit function and information processing capabilities of cortical neurons, producing behavioral defects common in neurodevelopmental and psychiatric disease (Yizhar et al., 2011; Nelson and Valakh, 2015; Antoine et al., 2019; Sohal and Rubenstein, 2019). Such disturbances to the E-I ratio in neurodevelopment can be caused by compensatory homeostatic plasticity in response to genetic defects, such as in mouse models of fragile X syndrome (Antoine et al., 2019). Recent work implicates mitochondria as mediators of homeostatic plasticity, with more pronounced changes in the mitochondrial proteome in response to activity deprivation in mice modeling fragile X syndrome (Bülow et al., 2021). Given these findings and our results here reporting enriched expression of nuclear-encoded mitochondrial transcripts in PV interneurons, it is tempting to speculate that disturbances in mitochondrial composition contribute to altered E-I ratios common in disease.

The heterogeneity we observed in ribosomal and respiratory chain proteins, as measured in our proteomic data or predicted from the single-cell transcript datasets, can be interpreted in the following ways. First, differences in expression do not necessarily mean that the stoichiometries differ from what is expected of the respiratory chain complexes embedded in the inner mitochondrial membrane or the mitochondrial ribosome in the matrix of the organelle. These expression differences may reflect anatomic and cell type-specific regulation of free subunits in the cytoplasm before they are targeted to their corresponding mitochondrial compartments. This hypothesis would suggest that the biogenesis or destruction of respiratory complexes or the mitochondrial ribosome subunits is different among anatomic regions or cell types. A second model considers heterogeneity in the composition of these complexes, a possibility bolstered by recent findings of variable neuronal cytoplasmic ribosome composition (Fusco et al., 2021). The contributions of these models to the stochiometric assembly of these mitochondrial complexes awaits further experimentation.

Our analysis of expression of SLC25A transporters showed that, as predicted, this family of proteins has variable expression across regions and cell types (Figs. 4, 5). Interestingly, we found that several SLC25A transporters were expressed at higher levels in small populations of cells (Fig. 5). The increased expression of SLC25A family members in distinct cell populations included multiple orphan transporters whose function is unclear, such as SLC25A30, SLC25A34, and SLC25A39 (Palmieri, 2013; Palmieri and Monné, 2016; Palmieri et al., 2020), and transporters linked to neurodevelopmental disorders, such as the citrate transporter SLC25A1 and those annotated in the SFARI database of autism spectrum-linked genes (Fig. 5*B*). Mutations in SLC25A1 cause a rare, often fatal metabolic disorder characterized by neonatal epileptic encephalopathy (Nota et al., 2013). Moreover, SLC25A1 is part of the chromosomal interval deleted in 22q11.2 deletion syndrome, which is associated with increased risk for myriad neurodevelopmental disorders, most prominently schizophrenia (McDonald-McGinn et al., 2015). Recent work suggests that SLC25A1 and SLC25A4 are hub genes in the network of the perturbed brain proteome associated with 22q11.2 deletion syndrome (Gokhale et al., 2019). The distinctive expression patterns of SLC25A transporters that we observed may be an intrinsic cell autonomous characteristic defining a distinct cell population. Alternatively, such discrete cell populations may represent a transient metabolic state triggered by an acute stimulus. While we cannot resolve between these hypotheses until single-cell metabolomics is possible, genetically encoded biosensors for metabolites are an alternative tool that could be used to discriminate between these hypotheses. Subcellularly targeted and genetically encoded biosensors for lactate, glucose, ATP, NADH, and pyruvate have been successfully used in neurons, while biosensors for the TCA cycle metabolites citrate and α-ketoglutarate have also recently become available (Lüddecke et al., 2017; Koveal et al., 2020; Zhao et al., 2020). These tools can be used to support further investigation into our results here. Together, our results suggest that further investigation into the roles of the SLC25A transporter family in the brain will produce important insights into how mitochondria influence brain function and neurodevelopment. Our work provides a resource of several mitochondrial genes enriched in certain cell types or regions that will serve as a novel tool to inspire hypothesis generation and functional studies of mitochondrial regional and cellular heterogeneity in the brain.

## Synthesis

Reviewing Editor: Katalin Toth, University of Ottawa Decisions are customarily a result of the Reviewing Editor and the peer reviewers coming together and discussing their recommendations until a consensus is reached. When revisions are invited, a fact-based synthesis statement explaining their decision and outlining what is needed to prepare a revision will be listed below. The following reviewer(s) agreed to reveal their identity: Csaba Cserép.

This study offers novel and important information about mitochondrial diversity. Both reviewers were very positive about the importance of the data, however, there are few issues that need to be clarified. The authors need to provide a clear comparison with previously published data and elaborate on the potential reasons for the observed differences. It would also be important to validate the skewed data distribution to exclude potential methodological artifacts. In addition, both reviewers provided several points on how the authors should increase the clarity of the data presentation throughout the manuscript.

Rev.#1

Overall, I find this an interesting paper, as it clarifies to some degree that cell type-specific changes underlie many of the brain region-specific differences in mitochondrial composition. Also, the result that relatively core component of the mitochondria are divers even between different neuronal cell classes comes a bit as a surprise to me. At the same time, there is now already firm evidence that mitochondrial composition differs substantially between cell types, including in the CNS, so that most people will assume that indeed most brain region-specific differences will be explained by cell type composition differences. Moreover, the authors re-measured omics data that have been recorded before, in some cases with higher coverage - so there is limited novelty here, and the new data need to be referenced against the published ones, and where differences are apparent further experiments are needed to resolve such discrepancies.

Therefore, I have a number of concerns that will need further data, analyses and explanations:

1) Overall, I find the proteomics data in Fig. 2 somewhat surprising:

- The extent of differences between brain regions is remarkable - and such data have certainly been measured several times before, to my reading without such massive differences (e.g. by Sharma et al. Nat Neurosci 2015 published from the Mann and Simons labs; note also that coverage here seems quite a bit better with >11.000 proteins). Are these results consistent (e.g. the now reported >85% of proteins significantly changed between two brain proximate and related regions such as cortex and hippocampus vs. Fig. 5a/b in Sharma et al)?

- Why are the mitochondrial volcano blots so skewed in Fig. 2A2? How would the authors speculate this skewed proteome representation of mitochondrial components squares with the relatively symmetrical representation of transcripts (only for Hipp-Striat the volcanoes look somewhat similar, albeit the hits do not match)? I would suspect the explanation to be simply a difference in mitochondrial content of the isolates(which could be a feature of the brain regions or different degrees of non-mitochondrial contamination, e.g. derived from variable myelin content etc.). These results should be checked, e.g. with Western blots for some extreme hits (including Ndufaf2, the sole ‘outlier’ on the non-cortex side and some high cortex hits); moreover, these result can be easily compared to Sharma et al.. Should these original results prove to be an artifact, I would suggest attempting brain-region specific mitochondrial isolates as a source material for a further round of proteomic analysis.

- I am not sure what I am missing - but I cannot see the striatum data in Fig 2D.

2) Some figures are confusing to me. In Fig. 3 B vs. C-E (and similarly in Fig. 4); I assume the green color in C-E to be the GABAergic neurons vs. the glutamatergic neurons in gray. In B, I assume all colors from the scale on the right to be represented - but the graphs look similarly dominated by green and gray, at least on printouts. This would probably mean that some brain regions that are encoded in green are overrepresented in the data set (and the rest just mixes to gray). In any case, to avoid confusion, I recommend excluding the colors used for the two neuron groups from the brain region plots - and to ensure that a random mix of the brain region colors does not resemble a color used for the major neuron classes.

Rev.#2

The paper „Heterogeneous Expression of Nuclear Encoded Mitochondrial Genes Distinguishes Inhibitory and Excitatory Neurons” was evaluated. In the present study, the authors analyzed a large set of nuclear-encoded mitochondrial genes (whose products build up either fixed stoichiometry complexes like the respiratory chain elements, the mitochondrial ribosome, or proteins that function as monomers, like the members of the SLC25 transporter-family) through proteomics and transcriptomics of different brain regions and different neuronal populations. They found, that the protein expression of these nuclear encoded mitochondrial genes show differentiated expression profiles defining distinct regional clusters (cortex, hippocampus, and striatum), while the transcript expression of these genes could not distinguish between these three different brain regions. Interestingly, mitochondrial ribosome, SLC25A or respiratory chain complex transcripts could distinguish between these brain regions when analyzed at the single cell level. Finally, expression of mitochondrial genes differed significantly between excitatory and inhibitory neurons, as well as between different GABAergic interneuron subpopulations. The results confirm heterogeneous mitochondrial composition across brain regions and cell types, expanding our knowledge on brain region and cell-type specific mitochondrial heterogeneity. The experiments are well designed, the results are robust, and the authors utilize an elegant palette of methodologies. The study is timely, interesting and fits well in the Journal’s profile. The manuscript is well written, the relevant literature is cited. The figures are generally high quality, although some modifications are needed. The description of the methods is clear, the discussion is well balanced. I have only a few comments.

Minor points:

1. On Fig. 1. A1 some of the gene names overlayed onto the volcano plots are impossible to read. I would move these names a bit farther away from the dots, and would not stick to the idea of using only parallel segments connesting these texts and the corresponding dots.

2. On Fig. 1. A2 I would also label which side of the volcano plots correspond to which region, like on Fig. 1. A1.

3. On Fig. 2. A1 the “Enriched brain-region” texts overlap with the dashed lines and the data points sometimes. Please decrease font size.

4. On Fig. 2. A2 I would also label which side of the volcano plots correspond to which region, like on Fig. 2. A1.

5. The most important point is that the single point cell representation maps plotted from multidimensional mRNA expression data (used on Figures 3. B-E, 4. and 5.) should be explained in more detail. Readers - not expert in transcriptomics - might find it a bit puzzling to interpret these maps, I would suggest to extend the figure legend and/or the results section to explain these maps in more detail and a more simple way. Also the color-coding of different brain regions on Fig. 3. B and Fig. 4. is difficult to interpret, since from these 21 categories two or three (maximum four) can be distinguished on the maps.

## Author Response

June 25, 2021

Dr. Katalin Toth

Reviewing Editor

eNeuro

Dear Katalin,

We would like to thank you for supporting a resubmission of our manuscript

"Heterogeneous Expression of Nuclear Encoded Mitochondrial Genes Distinguishes Inhibitory

and Excitatory Neurons” (eNeuro eN-NWR-0232-21).

We thank reviewers for the encouraging comments about our work and for their

constructive suggestions and comments. We believe that we have assembled an improved

manuscript with the reviewers’ help.

Below, we provide a detailed response to Reviewers’ comments. We highlighted all text

changes in gray within the revised manuscript.

Reviewer 1.

Comment 1: “ Overall, I find the proteomics data in Fig. 2 somewhat surprising: - The extent of

differences between brain regions is remarkable - and such data have certainly been

measured several times before, to my reading without such massive differences (e.g. by

Sharma et al. Nat Neurosci 2015 published from the Mann and Simons labs; note also that

coverage here seems quite a bit better with >11.000 proteins). Are these results consistent (e.g.

the now reported >85% of proteins significantly changed between two brain proximate and

related regions such as cortex and hippocampus vs. Fig. 5a/b in Sharma et al)?......”

We thanks the reviewer for the comment and for suggesting to us a comparison of our

results with those of Sharma et al. To address this comment, we have taken the following

actions:

1.1. To answer how our findings match with those of Sharma, we added new

analyses in revised Figure 2. We found that our datasets overlap beyond what is

expected by chance. See Venn diagram inserts in revised figure 2B. For example, our

mouse cortex-enriched proteome captured 69.2% of the mouse motor cortex-enriched

proteome described by Sharma et al. [1]. This overlap is 30.8 times above what it is

expected by chance (p=7.9E-195, Figure 2B, Venn diagram inserts). In order to offer

further accessibility to our data, we have added revised extended data 2-1 where we list

all hits per brain region as well as mitochondrial hits by region.

1.2. Since the reviewer comment may be interpreted as skepticism about the

quality of our datasets, we have now included multiscatter plots, correlation matrices,

and PCA analyses that provide a comprehensive assessment of the quality of our data

(see revised Figure 2A). We think this new evidence shows that our TMT mass

spectrometry results abide to stringent standards. Therefore, if we accept the premise

that our data are technically sound, why do our data appear to be different from those

of Sharma et al? While Sharma’s study is seminal, it was performed using approaches

that differ from ours in several aspects that we would like to highlight as we believe

they explain the differences between studies. eNeuro eN-NWR-0232-21

2

2

1.2.1. A first point of divergence is the age of animals, we used mice

aged 40-42 days while Sharma used 60 days.

1.2.2. We used 5 biological replicates and Sharma used 3 biological

replicates, thus we are better powered to robustly detects differences in

expression.

1.2.3. We used microdissection punch biopsies as compared to Sharma

et al, who used whole brain areas.

1.2.4. Sharma et al. used LFQ mass spectrometry while our studies use

TMT. This is a critical distinction as TMT is better positioned to detect with high

precision differences in proteins expression between samples. Steven Gigy,

performing comparisons between LFQ and TMT stated: “the TMT method

detected changes that reached statistical significance three times more often

due to higher precision and fewer missing values” [2]. There is a growing

consensus in the literature about the better performance of TMT as compared

to LFQ [3, 4].

1.2.5. The reviewer point us toward Figures 5a/b in Sharma et al.

depicting a PCI analysis of brain regions and a heat map of differences. These

data in Sharma et al. tell us that their study has a higher variance in their

datasets as compared to ours. For example, the variance captured by PCI1 in

Sharma is 34.8% (Sharma et al. Fig. 5A) versus our study where PCI1 captures

97% variance (see revised Figure 2A). Also, see the heat map in Sharma et al. Fig.

5B where many of the rows are listed as “<4-fold expression and/or insignificant

difference”. We think these observations extracted from Figures 5a/b in

Sharma et al. likely emerge from the use of LFQ as compared to TMT.

In summary, since the publication from Sharma et al in 2015, we think we have

benefited from 6 years of technological improvements in mass spectrometry approaches plus a

better powered experimental design that captures more differences in between regions. We

believe that if the Mann and Simons labs were to perform these studies today they would

choose TMT. The explanations we provide in this letter are now included in the revised

manuscript text in an abridged manner.

Comment 2: “ Why are the mitochondrial volcano blots so skewed in Fig. 2A2? How

would the authors speculate this skewed proteome representation of mitochondrial

components squares with the relatively symmetrical representation of transcripts (only for

Hipp-Striat the volcanoes look somewhat similar, albeit the hits do not match)? I.”

We do not have an explanation for these differences. However, we would like to point

that it is now a recognized fact that comparing RNAseq and proteome data leads to poor

correlations between 0.2 to 0.5. This problem is a common occurrence in diverse tissues and cell

types, including the brain, with correlations between mRNA and protein expression below 0.5

[5-9]. We hope this explanation provides an answer to this apparent paradox between RNAseq

and proteome results.

Comment 3: “ These results should be checked, e.g. with Western blots for some

extreme hits (including Ndufaf2, the sole ‘outlier’ on the non-cortex side and some high cortex

hits); moreover, these result can be easily compared to Sharma et al.......”

The reviewer has raised an important point that we tried to address before the initial

submission. We focused on the most divergent SLC25A transporters and subunits of the eNeuro eN-NWR-0232-21

3

3

mitochondrial ribosome. Multiple commercial antibodies failed to perform satisfactorily

preventing us from conducting experiments as those suggested by the reviewer. Antibodies

against SLC25A transporters and mitochondrial ribosome subunits recognized multiple bands on

tissue or cell extracts. As a point of reference, it took us a year to get antibodies against SLC25A1

and MRPL40 that perform by immunoblot in brain tissue and that we could validate either in

CRISPR KO cells or against recombinant proteins. We hope that the reviewer will appreciate that

to address this comment about data validation, we will incur in a long and winding road

pursuing uncertain antibodies that may lead nowhere.

We would like to request a waiver of this request as systems biology papers increasingly

have abandoned immunoblot as a validation tool and taken either functional-anatomical

validations (see for example Sharma et al. [1]) or orthogonal omics datasets as validation

approaches [10-12]. In our case, the single cell RNAseq analysis is the approach we took to

validate our hypothesis derived from the proteome dataset.

Comment 4: “Should these original results prove to be an artifact, I would suggest

attempting brain-region specific mitochondrial isolates as a source material for a further

round of proteomic analysis"

This is a great suggestion yet we would like to remind the reviewer that in the present

manuscript we work with mouse microdissected tissue of ∼160 μm

3

in volume or ∼220 μg of wet

weight. We have performed experiments as those proposed by the reviewer. However, we can

only perform them with bulk dissected brain regions from 5-10 mice (manuscript under review).

Thus, a critical experimental design feature, microdissection, that captures regional differences

will be lost in attempts to purify mitochodria.

Comment 4 “ I am not sure what I am missing - but I cannot see the striatum data in

Fig 2D. “.

The striatum data in revised Figure 2F are depicted by purple circles that we have better

labeled. The five biological replicates are all overlapping thus appearing as just one circle.

Comment 4 “ 2) Some figures are confusing to me. In Fig. 3 B vs. C-E (and similarly in

Fig. 4); I assume the green color in C-E to be the GABAergic neurons vs. the glutamatergic

neurons in gray. In B, I assume all colors from the scale on the right to be represented - but the

graphs look similarly dominated by green and gray, at least on printouts. This would probably

mean that some brain regions that are encoded in green are overrepresented in the data set

(and the rest just mixes to gray). In any case, to avoid confusion, I recommend excluding the

colors used for the two neuron groups from the brain region plots - and to ensure that a

random mix of the brain region colors does not resemble a color used for the major neuron

classes. “.

We thank the reviewer for this suggestions. Figures have been amended as indicated.

Reviewer 2.

Comment 1: “ On Fig. 1. A1 some of the gene names overlayed onto the volcano plots are

impossible to read. I would move these names a bit farther away from the dots, and would not

stick to the idea of using only parallel segments connesting these texts and the corresponding

dots.”

We have amended the figure as suggested.eNeuro eN-NWR-0232-21

4

4

Comment 2: On Fig. 1. A2 I would also label which side of the volcano plots correspond to

which region, like on Fig. 1. A1.”

We have introduced the changes requested in the figure.

Comment 3: “On Fig. 2. A1 the “Enriched brain-region” texts overlap with the dashed lines

and the data points sometimes. Please decrease font size..”

We have changed the orientation of the text to achieved the outcome suggested by the

reviewer.

Comment 4: “On Fig. 2. A2 I would also label which side of the volcano plots correspond to

which region, like on Fig. 2. A1.”

We introduced the suggested modification.

Comment 5: “The most important point is that the single point cell representation maps

plotted from multidimensional mRNA expression data (used on Figures 3. B-E, 4. and 5.) should

be explained in more detail. Readers - not expert in transcriptomics - might find it a bit

puzzling to interpret these maps, I would suggest to extend the figure legend and/or the

results section to explain these maps in more detail and a more simple way..”

We thank the reviewer for this important suggestion. We have introduced text to

explain better the t-SNE atlases. We hope we achieve the goal requested by the reviewer.

Comment 6: “Also the color-coding of different brain regions on Fig. 3. B and Fig. 4. is difficult

to interpret, since from these 21 categories two or three (maximum four) can be distinguished

on the maps.”

We have changed the colors in these atlases as a way to find a solution to the issue

raised by the reviewer. However, we think is nearly impossible to address this request as the

graphs contains ∼70,000 data points distributed across ∼21 categories. We attempted other

color palettes without success. We hope the reviewer will waive this suggestion as multiple

efforts to fix this problem were unsuccessful.

References

1. Sharma, K., et al., Cell type- and brain region-resolved mouse brain proteome. Nat

Neurosci, 2015. 18(12): p. 1819-31.

2. O’Connell, J.D., et al., Proteome-Wide Evaluation of Two Common Protein Quantification

Methods. J Proteome Res, 2018. 17(5): p. 1934-1942.

3. Li, Z., et al., Systematic comparison of label-free, metabolic labeling, and isobaric

chemical labeling for quantitative proteomics on LTQ Orbitrap Velos. J Proteome Res,

2012. 11(3): p. 1582-90.

4. Ahrne, E., et al., Evaluation and Improvement of Quantification Accuracy in Isobaric

Mass Tag-Based Protein Quantification Experiments. J Proteome Res, 2016. 15(8): p.

2537-47.

5. Ghazalpour, A., et al., Comparative analysis of proteome and transcriptome variation in

mouse. PLoS Genet, 2011. 7(6): p. e1001393.

6. Wang, D., et al., A deep proteome and transcriptome abundance atlas of 29 healthy

human tissues. Mol Syst Biol, 2019. 15(2): p. e8503.eNeuro eN-NWR-0232-21

5

5

7. de Sousa Abreu, R., et al., Global signatures of protein and mRNA expression levels. Mol

Biosyst, 2009. 5(12): p. 1512-26.

8. Schwanhausser, B., et al., Global quantification of mammalian gene expression control.

Nature, 2011. 473(7347): p. 337-42.

9. Carlyle, B.C., et al., A multiregional proteomic survey of the postnatal human brain. Nat

Neurosci, 2017. 20(12): p. 1787-1795.

10. Seyfried, N.T., et al., A Multi-network Approach Identifies Protein-Specific Co-expression

in Asymptomatic and Symptomatic Alzheimer’s Disease. Cell Syst, 2017. 4(1): p. 60-72 e4.

11. Wingo, T.S., et al., Brain proteome-wide association study implicates novel proteins in

depression pathogenesis. Nat Neurosci, 2021. 24(6): p. 810-817.

12. Wingo, A.P., et al., Shared proteomic effects of cerebral atherosclerosis and Alzheimer’s

disease on the human brain. Nat Neurosci, 2020. 23(6): p. 696-700

## References

[B1] Abrahams BS, Arking DE, Campbell DB, Mefford HC, Morrow EM, Weiss LA, Menashe I, Wadkins T, Banerjee-Basu S, Packer A (2013) SFARI Gene 2.0: a community-driven knowledgebase for the autism spectrum disorders (ASDs). Mol Autism 4:36. 10.1186/2040-2392-4-36 24090431 PMC3851189

[B2] Afgan E, Baker D, Batut B, van den Beek M, Bouvier D, Cech M, Chilton J, Clements D, Coraor N, Grüning BA, Guerler A, Hillman-Jackson J, Hiltemann S, Jalili V, Rasche H, Soranzo N, Goecks J, Taylor J, Nekrutenko A, Blankenberg D (2018) The Galaxy platform for accessible, reproducible and collaborative biomedical analyses: 2018 update. Nucleic Acids Res 46:W537–W544. 10.1093/nar/gky379 29790989 PMC6030816

[B3] Andersen JS, Mann M (2006) Organellar proteomics: turning inventories into insights. EMBO Rep 7:874–879. 10.1038/sj.embor.7400780 16953200 PMC1559674

[B4] Andrews S (2010) FastQC: a quality control tool for high throughput sequence data. Available at http://www.bioinformatics.babraham.ac.uk/projects/fastqc/.

[B5] Anitha A, Nakamura K, Thanseem I, Yamada K, Iwayama Y, Toyota T, Matsuzaki H, Miyachi T, Yamada S, Tsujii M, Tsuchiya KJ, Matsumoto K, Iwata Y, Suzuki K, Ichikawa H, Sugiyama T, Yoshikawa T, Mori N (2012) Brain region-specific altered expression and association of mitochondria-related genes in autism. Mol Autism 3:12. 10.1186/2040-2392-3-12 23116158 PMC3528421

[B6] Antoine MW, Langberg T, Schnepel P, Feldman DE (2019) Increased excitation-inhibition ratio stabilizes synapse and circuit excitability in four autism mouse models. Neuron 101:648–661.e4. 10.1016/j.neuron.2018.12.026 30679017 PMC6733271

[B7] Aryaman J, Johnston IG, Jones NS (2018) Mitochondrial heterogeneity. Front Genet 9:718. 10.3389/fgene.2018.00718 30740126 PMC6355694

[B8] Attwell D, Laughlin SB (2001) An energy budget for signaling in the grey matter of the brain. J Cereb Blood Flow Metab 21:1133–1145. 10.1097/00004647-200110000-00001 11598490

[B9] Barr JB, Somerville RA, Chung YL, Fraser JR (2004) Microdissection: a method developed to investigate mechanisms involved in transmissible spongiform encephalopathy pathogenesis. BMC Infect Dis 4:8. 10.1186/1471-2334-4-8 15053838 PMC375531

[B10] Bernstein BW, Bamburg JR (2003) Actin-ATP hydrolysis is a major energy drain for neurons. J Neurosci 23:1–6. 12514193 10.1523/JNEUROSCI.23-01-00002.2003 PMC6742122

[B11] Bülow P, Zlatic SA, Wenner PA, Bassell GJ, Faundez V (2021) FMRP attenuates activity dependent modifications in the mitochondrial proteome. Mol Brain 14:75. 10.1186/s13041-021-00783-w 33931071 PMC8086361

[B12] Cardoso-Moreira M, Halbert J, Valloton D, Velten B, Chen C, Shao Y, Liechti A, Ascenção K, Rummel C, Ovchinnikova S, Mazin PV, Xenarios I, Harshman K, Mort M, Cooper DN, Sandi C, Soares MJ, Ferreira PG, Afonso S, Carneiro M, et al. (2019) Gene expression across mammalian organ development. Nature 571:505–509. 10.1038/s41586-019-1338-5 31243369 PMC6658352

[B13] Carlyle BC, Kitchen RR, Kanyo JE, Voss EZ, Pletikos M, Sousa AMM, Lam TT, Gerstein MB, Sestan N, Nairn AC (2017) A multiregional proteomic survey of the postnatal human brain. Nat Neurosci 20:1787–1795. 10.1038/s41593-017-0011-2 29184206 PMC5894337

[B14] Chinnery PF (1993) Mitochondrial disorders overview. In: GeneReviews (Adam MP, Ardinger HH, Pagon RA, Wallace SE, Bean LJH, Mirzaa G, Amemiya A, eds). Seattle: University of Washington. 20301403

[B15] Cserép C, Pósfai B, Schwarcz AD, Dénes A (2018) Mitochondrial ultrastructure is coupled to synaptic performance at axonal release sites. eNeuro 5:ENEURO.0390-17.2018. 10.1523/ENEURO.0390-17.2018 PMC5788698 29383328

[B16] Cunningham CN, Rutter J (2020) 20,000 picometers under the OMM: diving into the vastness of mitochondrial metabolite transport. EMBO Rep 21:e50071. 10.15252/embr.202050071 32329174 PMC7202207

[B17] de Sousa Abreu R, Penalva LO, Marcotte EM, Vogel C (2009) Global signatures of protein and mRNA expression levels. Mol Biosyst 5:1512–1526. 10.1039/b908315d 20023718 PMC4089977

[B18] Erö C, Gewaltig MO, Keller D, Markram H (2018) A cell atlas for the mouse brain. Front Neuroinform 12:84. 10.3389/fninf.2018.00084 30546301 PMC6280067

[B19] Fecher C, Trovò L, Müller SA, Snaidero N, Wettmarshausen J, Heink S, Ortiz O, Wagner I, Kühn R, Hartmann J, Karl RM, Konnerth A, Korn T, Wurst W, Merkler D, Lichtenthaler SF, Perocchi F, Misgeld T (2019) Cell-type-specific profiling of brain mitochondria reveals functional and molecular diversity. Nat Neurosci 22:1731–1742. 10.1038/s41593-019-0479-z 31501572

[B20] Ferguson BR, Gao WJ (2018) PV interneurons: critical regulators of E/I balance for prefrontal cortex-dependent behavior and psychiatric disorders. Front Neural Circuits 12:37. 10.3389/fncir.2018.00037 29867371 PMC5964203

[B21] Fusco CM, Desch K, Dörrbaum AR, Wang M, Staab A, Chan ICW, Vail E, Villeri V, Langer JD, Schuman EM (2021) Neuronal ribosomes dynamically exchange ribosomal proteins in a context-dependent manner. bioRxiv 2021.2003.2025.437026. 10.1038/s41467-021-26365-x PMC8531293 34675203

[B22] Geiger T, Velic A, Macek B, Lundberg E, Kampf C, Nagaraj N, Uhlen M, Cox J, Mann M (2013) Initial quantitative proteomic map of 28 mouse tissues using the SILAC mouse. Mol Cell Proteomics 12:1709–1722. 10.1074/mcp.M112.024919 23436904 PMC3675825

[B23] Ghazalpour A, Bennett B, Petyuk VA, Orozco L, Hagopian R, Mungrue IN, Farber CR, Sinsheimer J, Kang HM, Furlotte N, Park CC, Wen PZ, Brewer H, Weitz K, Camp DG, Pan C, Yordanova R, Neuhaus I, Tilford C, Siemers N, et al. (2011) Comparative analysis of proteome and transcriptome variation in mouse. PLoS Genet 7:e1001393. 10.1371/journal.pgen.1001393 21695224 PMC3111477

[B24] Giurgiu M, Reinhard J, Brauner B, Dunger-Kaltenbach I, Fobo G, Frishman G, Montrone C, Ruepp A (2019) CORUM: the comprehensive resource of mammalian protein complexes-2019. Nucleic Acids Res 47:D559–D563. 10.1093/nar/gky973 30357367 PMC6323970

[B25] Gokce O, Stanley GM, Treutlein B, Neff NF, Camp JG, Malenka RC, Rothwell PE, Fuccillo MV, Südhof TC, Quake SR (2016) Cellular taxonomy of the mouse striatum as revealed by single-cell RNA-seq. Cell Rep 16:1126–1137. 10.1016/j.celrep.2016.06.059 27425622 PMC5004635

[B26] Gokhale A, Hartwig C, Freeman AAH, Bassell JL, Zlatic SA, Sapp Savas C, Vadlamudi T, Abudulai F, Pham TT, Crocker A, Werner E, Wen Z, Repetto GM, Gogos JA, Claypool SM, Forsyth JK, Bearden CE, Glausier J, Lewis DA, et al. (2019) Systems analysis of the 22q11.2 microdeletion syndrome converges on a mitochondrial interactome necessary for synapse function and behavior. J Neurosci 39:3561–3581. 10.1523/JNEUROSCI.1983-18.2019 30833507 PMC6495129

[B27] Gokhale A, Lee CE, Zlatic SA, Freeman AAH, Shearing N, Hartwig C, Ogunbona O, Bassell JL, Wynne ME, Werner E, Xu C, Wen Z, Doung D, Seyfried NT, Bearden CE, Oláh VJ, Rowan MJM, Glausier JR, Lewis DA, Faundez V (2021) Mitochondrial proteostasis requires genes encoded in a neurodevelopmental syndrome locus. J Neurosci. Advance online pubication. Retrieved Jul 14, 2021. doi: 10.1523/JNEUROSCI.2197-20.2021. PMC8336702 34261699

[B28] Gorman GS, Chinnery PF, DiMauro S, Hirano M, Koga Y, McFarland R, Suomalainen A, Thorburn DR, Zeviani M, Turnbull DM (2016) Mitochondrial diseases. Nat Rev Dis Primers 2:16080. 10.1038/nrdp.2016.80 27775730

[B29] Harris JJ, Jolivet R, Attwell D (2012) Synaptic energy use and supply. Neuron 75:762–777. 10.1016/j.neuron.2012.08.019 22958818

[B30] He P, Williams BA, Trout D, Marinov GK, Amrhein H, Berghella L, Goh ST, Plajzer-Frick I, Afzal V, Pennacchio LA, Dickel DE, Visel A, Ren B, Hardison RC, Zhang Y, Wold BJ (2020) The changing mouse embryo transcriptome at whole tissue and single-cell resolution. Nature 583:760–767. 10.1038/s41586-020-2536-x 32728245 PMC7410830

[B31] Higginbotham L, Ping L, Dammer EB, Duong DM, Zhou M, Gearing M, Hurst C, Glass JD, Factor SA, Johnson ECB, Hajjar I, Lah JJ, Levey AI, Seyfried NT (2020) Integrated proteomics reveals brain-based cerebrospinal fluid biomarkers in asymptomatic and symptomatic Alzheimer’s disease. Sci Adv 6:eaaz9360. 33087358 10.1126/sciadv.aaz9360 PMC7577712

[B32] Inan M, Zhao M, Manuszak M, Karakaya C, Rajadhyaksha AM, Pickel VM, Schwartz TH, Goldstein PA, Manfredi G (2016) Energy deficit in parvalbumin neurons leads to circuit dysfunction, impaired sensory gating and social disability. Neurobiol Dis 93:35–46. 10.1016/j.nbd.2016.04.004 27105708

[B33] Ioannou MS, Jackson J, Sheu SH, Chang CL, Weigel AV, Liu H, Pasolli HA, Xu CS, Pang S, Matthies D, Hess HF, Lippincott-Schwartz J, Liu Z (2019) Neuron-astrocyte metabolic coupling protects against activity-induced fatty acid toxicity. Cell 177:1522–1535.e14. 10.1016/j.cell.2019.04.001 31130380

[B34] Kann O, Kovács R (2007) Mitochondria and neuronal activity. Am J Physiol Cell Physiol 292:C641–657. 10.1152/ajpcell.00222.2006 17092996

[B35] Kim D, Langmead B, Salzberg SL (2015) HISAT: a fast spliced aligner with low memory requirements. Nat Methods 12:357–360. 10.1038/nmeth.3317 25751142 PMC4655817

[B36] Kobak D, Berens P (2019) The art of using t-SNE for single-cell transcriptomics. Nat Commun 10:5416. 10.1038/s41467-019-13056-x 31780648 PMC6882829

[B37] Koveal D, Díaz-García CM, Yellen G (2020) Fluorescent biosensors for neuronal metabolism and the challenges of quantitation. Curr Opin Neurobiol 63:111–121. 10.1016/j.conb.2020.02.011 32559637 PMC7646541

[B38] Lepagnol-Bestel AM, Maussion G, Boda B, Cardona A, Iwayama Y, Delezoide AL, Moalic JM, Muller D, Dean B, Yoshikawa T, Gorwood P, Buxbaum JD, Ramoz N, Simonneau M (2008) SLC25A12 expression is associated with neurite outgrowth and is upregulated in the prefrontal cortex of autistic subjects. Mol Psychiatry 13:385–397. 10.1038/sj.mp.4002120 18180767

[B39] Liao Y, Smyth GK, Shi W (2014) featureCounts: an efficient general purpose program for assigning sequence reads to genomic features. Bioinformatics 30:923–930. 10.1093/bioinformatics/btt656 24227677

[B40] Love MI, Huber W, Anders S (2014) Moderated estimation of fold change and dispersion for RNA-seq data with DESeq2. Genome Biol 15:550. 10.1186/s13059-014-0550-8 25516281 PMC4302049

[B41] Lüddecke J, Francois L, Spät P, Watzer B, Chilczuk T, Poschet G, Hell R, Radlwimmer B, Forchhammer K (2017) PII protein-derived FRET sensors for quantification and live-cell imaging of 2-oxoglutarate. Sci Rep 7:1437. 10.1038/s41598-017-01440-w 28469248 PMC5431102

[B42] Mann K, Deny S, Ganguli S, Clandinin TR (2021) Coupling of activity, metabolism and behaviour across the *Drosophila* brain. Nature 593:244–248. 10.1038/s41586-021-03497-0 33911283 PMC10544789

[B43] Mattson MP, Gleichmann M, Cheng A (2008) Mitochondria in neuroplasticity and neurological disorders. Neuron 60:748–766. 10.1016/j.neuron.2008.10.010 19081372 PMC2692277

[B44] McDonald-McGinn DM, Sullivan KE, Marino B, Philip N, Swillen A, Vorstman JA, Zackai EH, Emanuel BS, Vermeesch JR, Morrow BE, Scambler PJ, Bassett AS (2015) 22q11.2 deletion syndrome. Nat Rev Dis Primers 1:15071. 10.1038/nrdp.2015.71 27189754 PMC4900471

[B45] Murgia M, Nagaraj N, Deshmukh AS, Zeiler M, Cancellara P, Moretti I, Reggiani C, Schiaffino S, Mann M (2015) Single muscle fiber proteomics reveals unexpected mitochondrial specialization. EMBO Rep 16:387–395. 10.15252/embr.201439757 25643707 PMC4364878

[B46] Nelson SB, Valakh V (2015) Excitatory/inhibitory balance and circuit homeostasis in autism spectrum disorders. Neuron 87:684–698. 10.1016/j.neuron.2015.07.033 26291155 PMC4567857

[B47] Nota B, Struys EA, Pop A, Jansen EE, Fernandez Ojeda MR, Kanhai WA, Kranendijk M, van Dooren SJM, Bevova MR, Sistermans EA, Nieuwint AWM, Barth M, Ben-Omran T, Hoffmann GF, de Lonlay P, McDonald MT, Meberg A, Muntau AC, Nuoffer JM, Parini R, et al. (2013) Deficiency in SLC25A1, encoding the mitochondrial citrate carrier, causes combined D-2- and L-2-hydroxyglutaric aciduria. Am J Hum Genet 92:627–631. 10.1016/j.ajhg.2013.03.009 23561848 PMC3617390

[B48] O’Connell JD, Paulo JA, O’Brien JJ, Gygi SP (2018) Proteome-wide evaluation of two common protein quantification methods. J Proteome Res 17:1934–1942. 10.1021/acs.jproteome.8b00016 29635916 PMC5984592

[B49] Pagliarini DJ, Calvo SE, Chang B, Sheth SA, Vafai SB, Ong SE, Walford GA, Sugiana C, Boneh A, Chen WK, Hill DE, Vidal M, Evans JG, Thorburn DR, Carr SA, Mootha VK (2008) A mitochondrial protein compendium elucidates complex I disease biology. Cell 134:112–123. 10.1016/j.cell.2008.06.016 18614015 PMC2778844

[B50] Palmieri F (2013) The mitochondrial transporter family SLC25: identification, properties and physiopathology. Mol Aspects Med 34:465–484. 10.1016/j.mam.2012.05.005 23266187

[B51] Palmieri F, Monné M (2016) Discoveries, metabolic roles and diseases of mitochondrial carriers: a review. Biochim Biophys Acta 1863:2362–2378. 10.1016/j.bbamcr.2016.03.007 26968366

[B52] Palmieri F, Scarcia P, Monné M (2020) Diseases caused by mutations in mitochondrial carrier genes SLC25: a review. Biomolecules 10:655. 10.3390/biom10040655 PMC7226361 32340404

[B53] Perez-Riverol Y, Csordas A, Bai J, Bernal-Llinares M, Hewapathirana S, Kundu DJ, Inuganti A, Griss J, Mayer G, Eisenacher M, Pérez E, Uszkoreit J, Pfeuffer J, Sachsenberg T, Yilmaz S, Tiwary S, Cox J, Audain E, Walzer M, Jarnuczak AF, et al. (2019) The PRIDE database and related tools and resources in 2019: improving support for quantification data. Nucleic Acids Res 47:D442–D450. 10.1093/nar/gky1106 30395289 PMC6323896

[B54] Ping L, Duong DM, Yin L, Gearing M, Lah JJ, Levey AI, Seyfried NT (2018) Global quantitative analysis of the human brain proteome in Alzheimer’s and Parkinson’s disease. Sci Data 5:180036. 10.1038/sdata.2018.36 29533394 PMC5848788

[B55] Ping L, Kundinger SR, Duong DM, Yin L, Gearing M, Lah JJ, Levey AI, Seyfried NT (2020) Global quantitative analysis of the human brain proteome and phosphoproteome in Alzheimer’s disease. Sci Data 7:315. 10.1038/s41597-020-00650-8 32985496 PMC7522715

[B56] Rath S, Sharma R, Gupta R, Ast T, Chan C, Durham TJ, Goodman RP, Grabarek Z, Haas ME, Hung WHW, Joshi PR, Jourdain AA, Kim SH, Kotrys AV, Lam SS, McCoy JG, Meisel JD, Miranda M, Panda A, Patgiri A, et al. (2021) MitoCarta3.0: an updated mitochondrial proteome now with sub-organelle localization and pathway annotations. Nucleic Acids Res 49:D1541–D1547. 10.1093/nar/gkaa1011 33174596 PMC7778944

[B57] Sanz-Morello B, Pfisterer U, Winther Hansen N, Demharter S, Thakur A, Fujii K, Levitskii SA, Montalant A, Korshunova I, Mammen PP, Kamenski P, Noguchi S, Aldana BI, Hougaard KS, Perrier JF, Khodosevich K (2020) Complex IV subunit isoform COX6A2 protects fast-spiking interneurons from oxidative stress and supports their function. EMBO J 39:e105759. 10.15252/embj.2020105759 32744742 PMC7507454

[B58] Schwanhäusser B, Busse D, Li N, Dittmar G, Schuchhardt J, Wolf J, Chen W, Selbach M (2011) Global quantification of mammalian gene expression control. Nature 473:337–342. 10.1038/nature10098 21593866

[B59] Segurado R, Conroy J, Meally E, Fitzgerald M, Gill M, Gallagher L (2005) Confirmation of association between autism and the mitochondrial aspartate/glutamate carrier SLC25A12 gene on chromosome 2q31. Am J Psychiatry 162:2182–2184. 10.1176/appi.ajp.162.11.2182 16263864

[B60] Sharma K, Schmitt S, Bergner CG, Tyanova S, Kannaiyan N, Manrique-Hoyos N, Kongi K, Cantuti L, Hanisch UK, Philips MA, Rossner MJ, Mann M, Simons M (2015) Cell type- and brain region-resolved mouse brain proteome. Nat Neurosci 18:1819–1831. 10.1038/nn.4160 26523646 PMC7116867

[B61] Sohal VS, Rubenstein JLR (2019) Excitation-inhibition balance as a framework for investigating mechanisms in neuropsychiatric disorders. Mol Psychiatry 24:1248–1257. 10.1038/s41380-019-0426-0 31089192 PMC6742424

[B62] Spinelli JB, Haigis MC (2018) The multifaceted contributions of mitochondria to cellular metabolism. Nat Cell Biol 20:745–754. 10.1038/s41556-018-0124-1 29950572 PMC6541229

[B63] Tasic B, Menon V, Nguyen TN, Kim TK, Jarsky T, Yao Z, Levi B, Gray LT, Sorensen SA, Dolbeare T, Bertagnolli D, Goldy J, Shapovalova N, Parry S, Lee C, Smith K, Bernard A, Madisen L, Sunkin SM, Hawrylycz M, et al. (2016) Adult mouse cortical cell taxonomy revealed by single cell transcriptomics. Nat Neurosci 19:335–346. 10.1038/nn.4216 26727548 PMC4985242

[B64] Tasic B, Yao Z, Graybuck LT, Smith KA, Nguyen TN, Bertagnolli D, Goldy J, Garren E, Economo MN, Viswanathan S, Penn O, Bakken T, Menon V, Miller J, Fong O, Hirokawa KE, Lathia K, Rimorin C, Tieu M, Larsen R, et al. (2018) Shared and distinct transcriptomic cell types across neocortical areas. Nature 563:72–78. 10.1038/s41586-018-0654-5 30382198 PMC6456269

[B65] Thomas CI, Keine C, Okayama S, Satterfield R, Musgrove M, Guerrero-Given D, Kamasawa N, Young SM Jr (2019) Presynaptic mitochondria volume and abundance increase during development of a high-fidelity synapse. J Neurosci 39:7994–8012. 10.1523/JNEUROSCI.0363-19.2019 31455662 PMC6786813

[B66] Vafai SB, Mootha VK (2012) Mitochondrial disorders as windows into an ancient organelle. Nature 491:374–383. 10.1038/nature11707 23151580

[B67] Voogd J, Glickstein M (1998) The anatomy of the cerebellum. Trends Neurosci 21:370–375. 10.1016/s0166-2236(98)01318-6 9735944

[B68] Wang D, Eraslan B, Wieland T, Hallström B, Hopf T, Zolg DP, Zecha J, Asplund A, Li LH, Meng C, Frejno M, Schmidt T, Schnatbaum K, Wilhelm M, Ponten F, Uhlen M, Gagneur J, Hahne H, Kuster B (2019) A deep proteome and transcriptome abundance atlas of 29 healthy human tissues. Mol Syst Biol 15:e8503. 10.15252/msb.20188503 30777892 PMC6379049

[B69] Werner T, Becher I, Sweetman G, Doce C, Savitski MM, Bantscheff M (2012) High-resolution enabled TMT 8-plexing. Anal Chem 84:7188–7194. 10.1021/ac301553x 22881393

[B70] Wheeler DW, White CM, Rees CL, Komendantov AO, Hamilton DJ, Ascoli GA (2015) Hippocampome.org: a knowledge base of neuron types in the rodent hippocampus. Elife 4:e09960. 10.7554/eLife.09960 26402459 PMC4629441

[B71] Wilhelm M, Schlegl J, Hahne H, Gholami AM, Lieberenz M, Savitski MM, Ziegler E, Butzmann L, Gessulat S, Marx H, Mathieson T, Lemeer S, Schnatbaum K, Reimer U, Wenschuh H, Mollenhauer M, Slotta-Huspenina J, Boese JH, Bantscheff M, Gerstmair A, et al. (2014) Mass-spectrometry-based draft of the human proteome. Nature 509:582–587. 10.1038/nature13319 24870543

[B72] Yao Z, van Velthoven CTJ, Nguyen TN, Goldy J, Sedeno-Cortes AE, Baftizadeh F, Bertagnolli D, Casper T, Chiang M, Crichton K, Ding SL, Fong O, Garren E, Glandon A, Gouwens NW, Gray J, Graybuck LT, Hawrylycz MJ, Hirschstein D, Kroll M, et al. (2020) A taxonomy of transcriptomic cell types across the isocortex and hippocampal formation. Cell 184:3222–3241. 10.1016/j.cell.2021.04.021 PMC8195859 34004146

[B73] Yizhar O, Fenno LE, Prigge M, Schneider F, Davidson TJ, O’Shea DJ, Sohal VS, Goshen I, Finkelstein J, Paz JT, Stehfest K, Fudim R, Ramakrishnan C, Huguenard JR, Hegemann P, Deisseroth K (2011) Neocortical excitation/inhibition balance in information processing and social dysfunction. Nature 477:171–178. 10.1038/nature10360 21796121 PMC4155501

[B74] Zeisel A, Muñoz-Manchado AB, Codeluppi S, Lönnerberg P, La Manno G, Juréus A, Marques S, Munguba H, He L, Betsholtz C, Rolny C, Castelo-Branco G, Hjerling-Leffler J, Linnarsson S (2015) Brain structure. Cell types in the mouse cortex and hippocampus revealed by single-cell RNA-seq. Science 347:1138–1142. 10.1126/science.aaa1934 25700174

[B75] Zhao Y, Shen Y, Wen Y, Campbell RE (2020) High-performance intensiometric direct- and inverse-response genetically encoded biosensors for citrate. ACS Cent Sci 6:1441–1450. 10.1021/acscentsci.0c00518 32875085 PMC7453566

